# SALL4 promotes cancer stem-like cell phenotype and radioresistance in oral squamous cell carcinomas via methyltransferase-like 3-mediated m6A modification

**DOI:** 10.1038/s41419-024-06533-9

**Published:** 2024-02-14

**Authors:** Junhong Huang, Huan Li, Zihui Yang, Rong Liu, Yahui Li, Yating Hu, Shengnan Zhao, Xiang Gao, Xinjie Yang, Jianhua Wei

**Affiliations:** State Key Laboratory of Oral & Maxillofacial Reconstruction and Regeneration, National Clinical Research Center for Oral Diseases, Shaanxi Key Laboratory of Stomatology, Department of Oral & Maxillofacial Surgery, School of Stomatology, Fourth Military Medical University, Xi’an, 710032 China

**Keywords:** Oral cancer, Cancer stem cells, Prognostic markers

## Abstract

Radioresistance imposes a great challenge in reducing tumor recurrence and improving the clinical prognosis of individuals having oral squamous cell carcinoma (OSCC). OSCC harbors a subpopulation of CD44(+) cells that exhibit cancer stem-like cell (CSC) characteristics are involved in malignant tumor phenotype and radioresistance. Nevertheless, the underlying molecular mechanisms in CD44( + )-OSCC remain unclear. The current investigation demonstrated that methyltransferase-like 3 (METTL3) is highly expressed in CD44(+) cells and promotes CSCs phenotype. Using RNA-sequencing analysis, we further showed that Spalt-like transcription factor 4 (SALL4) is involved in the maintenance of CSCs properties. Furthermore, the overexpression of SALL4 in CD44( + )-OSCC cells caused radioresistance in vitro and in vivo. In contrast, silencing SALL4 sensitized OSCC cells to radiation therapy (RT). Mechanistically, we illustrated that SALL4 is a direct downstream transcriptional regulation target of METTL3, the transcription activation of SALL4 promotes the nuclear transport of β-catenin and the expression of downstream target genes after radiation therapy, there by activates the Wnt/β-catenin pathway, effectively enhancing the CSCs phenotype and causing radioresistance. Herein, this study indicates that the METTL3/SALL4 axis promotes the CSCs phenotype and resistance to radiation in OSCC via the Wnt/β-catenin signaling pathway, and provides a potential therapeutic target to eliminate radioresistant OSCC.

## Introduction

Oral squamous cell carcinoma (OSCC) is the most common type of head and neck malignancies and is associated with a high recurrence rate and resistance to conventional radiotherapy, resulting in a poor prognosis for most patients with OSCC [1]. An important therapeutic option for treating patients with OSCC is radiation therapy (RT), which effectively kills cancer cells or reduces the extent of surgical resection [2], especially in patients with advanced tumors, multiple metastases, and the presence of adverse prognostic factors, such as vascular nerve invasion. However, not all patients with OSCC are sensitive to ionizing radiation; a few of them show clinical resistance to radiation therapy, a critical cause for the treatment failure and recurrence and metastasis of OSCC [3]. Therefore, a detailed and better mechanistic understanding of molecular mechanisms involved in causing radioresistance in OSCC is essential to enhance the prognosis of individuals with OSCC.

In approximately 50% of all malignancies, including OSCC, RT is found to be useful, and its efficacy depends on the sensitivity of the body to radiation and many biological processes within the tumor, such as evasion apoptosis, hypoxia and inflammation, immune evasion, repopulation by cancer stem cells (CSCs), enhanced DNA damage response, and altered cell cycle, et al. [4]. CSCs is a subpopulation that can renew itself and can also initiate tumor formation, and this subpopulation has also been associated with RT failure and recurrences [5]. Although reversing the radioresistance by targeting CSCs has been attempted, specific molecular regulatory events that occur in OSCC remain unclear.

N6-methyladenosine (m6A) is the most abundant modification in mRNA. This process is regulated by several enzymes, such as m6A methyltransferases, demethylases, and readers [6]. The m6A methyltransferase complex contains several enzymes (METTL3, METTL14, WTAP, and RBM15B). METTL3, which is the core of the m6A methyltransferases complex, plays a key catalytic role during the methylation reaction [7, 8]. The demethylation enzymes, FTO and ALKBH5, regulate the reversible m6A modification process. In addition, m6A-binding proteins, including the YTH and IGF2BP family proteins, HNRNPA2B1, and HNRNPC are also involved in this process [9]. Some investigations provided evidences on how m6A is basically involved in the maintenance of CSCs properties. A study showed that the METTL3/14 complex silencing significantly improved the glioblastoma stem cells (GSCs)-related self-renewal properties, resultantly leading to tumor progression. However, when METTL3 was overexpressed or FTO was silenced, GSCs was inhibited the maintenance of stemness [10]. These results hinted at the role of m6A in regulating CSCs. A separate study demonstrated that m6A modification mediated by IGF2BP2-binding protein stabilized long non-coding RNA DANCR contributed toward pancreatic cancer malignancy and enhanced CSCs stemness [11]. In addition, m6A modification is known to impose regulatory effects on stem cells of colorectal cancer, osteosarcoma, bladder cancer, and lung cancer [12]. Considering these results, there is a possibility that m6A modification is also closely related to the maintenance of CSCs in OSCC and tumor progression.

Previously, we successfully established an OSCC radioresistant cell line [13]; *SALL4*, a differentially expressed gene (DEG) associated with CSCs stemness, was screened from parental and radioresistant cells, by RNA-seq. The SALL4 gene, which is part of the Spalt-like gene family of transcription factors, is essential for embryonic stem cell maintenance of self-renewal and pluripotency. It has been linked to poor prognosis because of its overexpression in several different types of cancer [14]. It has also been shown that the SALL4 protein regulates gene expression and tumor progression through epigenetic modifications [15]. Hence, SALL4 has a key oncogenic function. Since SALL4 is associated closely with CSCs stemness and CSCs have radioresistant properties, we hypothesized that m6A-driven modulation of SALL4 expression may be involved in causing radioresistance in OSCC.

In present study, we found that METTL3 promoted CSCs stemness, and SALL4 overexpression enhanced OSCC radioresistance. We propose that the METTL3/SALL4 axis mediates the CSCs phenotype, causing radioresistance in OSCC through the signaling pathway of Wnt/β-catenin. SALL4 can be an attractive target for controlling radiotherapy resistance in OSCC.

## Materials and methods

### Cell culture and lines

We obtained the SCC9, SCC15, and CAL27 OSCC cell lines from the American Type Culture Collection (ATCC, USA). Both SCC4 cell lines and human oral epithelial cell (HOEC) were purchased from the China Center for Type Culture Collection. Previous work established the establishment of OSCC cell lines that are radioresistant (SCC9-RR and CAL27-RR) [13]. All cell lines were checked for mycoplasma contamination through short tandem repeat (STR) profiling before all studies were performed. Cells were cultured in a 37 °C, 5% CO_2_ incubator with DMEM/F12 medium (Gibco, USA) supplemented with 10%fetal bovine serum (FBS), streptomycin (100 μg/mL), and penicillin (100 μg/mL).

### Magnetic cell sorting

Cell suspensions were treated in a sorting buffer, which contained MACS Buffer A (autoMACS^®^ Rinsing Solution) and Buffer B (MACS^®^ BSA Stock Solution) in a 20:1 ratio. Centrifuge cell suspensions at 1000 *g* for 10 min. Add 20 µL of CD44 Microbeads (MiltenyiBiotec, Bergisch Galdbach, Germany) and 80 µL of sorting buffer per 1 × 10^7^ total cells. Mix well and incubated at 4 °C for 15 min in the dark, followed by MACS buffer washes and centrifugation for 10 min at 1000 rpm. Cells were suspended in 500 µL of sorting buffer per 1 × 10^7^ total cells. CD44(+) cells and CD44(-) cells were dissociated using a magnetic cell sorting device (MiltenyiBiotec, Bergisch Galdbach, Germany). Briefly, the CD44(+) cells are magnetically labeled with CD44 microbead before they were run through an LS magnetic column, CD44(+) cells were eluted when the magnet was taken away from the column. In addition, to prevent the differentiation of CD44(+) cells into CD44(-) cells after multiple passages, second-generation CD44(+) cells were used in all the experiments, i.e., after sorting, the CD44(+) cells were passaged only once to maintain their cancer stem cell properties.

### Irradiation

Via a medical linear accelerator (Varian, MA, USA), CD44( + )-OSCC cells or CD44(-)-OSCC cells were irradiated at varying doses (Gy) under the specific conditions: Dose rate = 2 Gy/min, SSD = 100 cm, irradiation field = 25 cm × 25 cm, and vertical irradiation. The cell culture flask was covered with a 5 cm dose compensation plates and placed in the middle of the irradiation field.

### Sequencing and bioinformatic analysis

Sequencing was performed to analyze DEGs of parental and radioresistant cells, with analysis of significance using the EdgeR package at a cutoff of |log2(fold change)| >1 and P < 0.05. Heat map was used to represent genes showing a significant change and those associated with the maintenance of CSCs phenotype. The database of Cancer Genome Atlas (TCGA) (portal.gdc.cancer.gov) of 330 tumors and 32 normal samples were used to obtain OSCC mRNA sequencing data. Moreover, METTL3-linked patient survival was evaluated via the GEPIA platform (gepia.cancer-pku.cn). The UALCAN database (ualcan.path.uab.edu) was approached for analyzing the lymph node and tumor metastasis. The correlation between CSCs-related genes and SALL4 mRNA in OSCC was evaluated using the GEO data set (www.ncbi.nlm.nih.gov/geo).

### Quantitative real-time PCR

All OSCC cell lines, as well as CD44( + )-OSCC and CD44(-)-OSCC cells, total RNA was extracted with the use of the TaKaRa MiniBEST Universal RNA Extraction kit (Cat#9767, TaKaRa, Inc., Otsu, Japan). The Epoch^TM^ spectrophotometer (BIO-TEK, Vermont, USA) was used to measure the concentration and purity of the RNA. Following the manufacturer’s instructions, cDNA was synthesized via a PrimeScript^TM^ RT Master Mix (Perfect Real Time) kit (Cat# RR036A, TaKaRa, Inc., Otsu, Japan). For quantitative real-time PCR, we utilized the TB Green Premix Ex Taq^TM^ II kit (Cat# RR820A, TaKaRa, Inc., Otsu, Japan). The 7500 Real-Time PCR System (Applied Biosystems, Carlsbad, CA, USA) was employed for gene expression detection. The relative expression of target genes was determined via the 2^-△△CT^ method, with GAPDH serving as an internal reference. Sangon Biotechnology Inc. (Shanghai, China) produced the primers, which are listed in Supplementary Table 1.

### Western blotting

Radioimmunoprecipitation assay (RIPA) buffer (Beyotime; Shanghai, China) containing protease and phosphatase inhibitors were utilized for extracting total protein from each sample. The BCA protein assay kit (Beyotime, Shanghai, China) was approached for determining the protein concentration in the samples. SDS-PAGE (4–12%) was employed for separating proteins (20 µg/well), and the separated proteins were then transferred to PVDF membranes (Millipore; Billerica, MA, USA). The membranes were then incubated with the primary antibody (Supplementary Table 2) overnight after being blocked in 5% skim milk in TBST for 1 h. Proteins were detected via a Chemi-Doc^TM^XRS + Image Lab^TM^ system (BIO-RAD; Thermo, USA) after incubation with goat anti-rabbit IgG H&L (HRP) (Abcam, Cambridge, UK, ab6721, 1:5000) secondary antibody at 37 °C for 1 h. Protein bands were analyzed with enhanced chemiluminescence (ECL) detection reagent. The western blot original data are listed in the Supplementary Material.

### Knockdown or overexpression of target genes

Cells were transfected with plasmids and siRNAs from Sangon Biotechnology Inc. (Shanghai, China) via Lipofectamine 2000 (Invitrogen, Carlsbad, CA, USA) according to the manufacturer’s procedure. qRT-PCR and western blotting were employed for analyzing the level of transfection success. After 48 and 72 h of transfection, mRNA and protein expression levels were measured. The siRNA primers tested in this work are detailed in Supplementary Table 1. Stably transfected cell lines with METTL3 knockdown and SALL4 overexpression were constructed by Hanheng Biological Company (Shanghai, China).

### Sphere formation assay

Spheroid media, which consisted of DMEM/F12 (Gibco, USA), 2×N2 supplements (Gibco, USA), 1×B27^®^supplements (Gibco, USA), insulin (5 μg/mL; Invitrogen, USA), bFGF (20 ng/mL; PeproTech, Rocky Hill, NJ, USA), and EGF (20 ng/mL; PeproTech, Cranbury, NJ, USA), were employed for cell culture. Cells were seeded at a density of 1000 cells per well in 24-well ultra-low attachment culture plates (PlatesCorning Inc., Corning, NY), The medium was added with fresh medium every 5 days, and then photographed using a microscope after 14 days of constant incubation in the same conditions.

### CD44^+^/ALDH1^+^ cell sorting

This experiment was conducted by setting up the following groups: blank control, CD44 (PE), ALDH1 (FITC), and CD44/ALDH1 double-stained, 1×10^6^ cells/mL were used. Since ALDH1 is an intracellular protein, membrane breaking was required for staining. First, the cells were incubated for 10 min at room temperature in a 500 μL pre-cooled fixative before centrifuged for 10 min at 1000 rpm. Subsequently, an appropriate amount of membrane-breaking reagent was added, and the samples were incubated for 10 min at room temperature before centrifuged at 1000 rpm for 10 min. Eventually, samples could be incubated with FITC-ALDH1A1 (Abcam, ab275646) and PE-CD44 (BioLegend, cat#338808) fluorescent antibodies at 4 °C for 30 min and detected using flow cytometry.

### MeRIP-qPCR assay

The m6A immunoprecipitation qPCR (MeRIP-qPCR) was performed using GenSeq^®^m6A MeRIP Kit (Cat No. GS-ET-001, Cloud-Seq Biotech, Shanghai, China). Briefly, RNA (1 µg/µL) was fragmented to a size of nearly 200 nt, and a portion of fragmented RNA (1–3 µg) was used as the input group, whereas the rest of the fragmented RNA was employed in immunoprecipitation experiment. Anti-m6A or anti-IgG antibody (2 µL) coated magnetic beads were utilized for immunoprecipitation. Purified RNA was then subjected to qPCR using SALL4-specific primers designed based on a motif-dependent m6A site predictor SRAMP (http://www.cuilab.cn/sramp) (Forward: GAGCTGTACTGAGCCACCAG; Reverse: GCATCCGGCTTCTCCTTCAT). Normalizing m6A enrichment could be achieved via the value generated using the input group as follows: %Input = 2^Ct [input] – Ct [IP]^ × Dilution multiplier × 100.

### Coimmunoprecipitation

The Rabbit anti-SALL4 (Proteintech Group, 24500-1-AP), Rabbit anti-METTL3 (Proteintech Group, 15073-1-AP) (10 μg) or IgG was added to the protein samples, which was incubated overnight at 4 °C. The antigen-antibody complex were then bound to Pierce^TM^ Protein A/G Agarose Beads (Thermo Fisher Scientific, Inc.) and incubated for overnight at 4 °C. After the supernatant was removed by centrifugation, proteins samples were incubated at 95 °C for 5 min and processed to western blotting analysis.

### Flow cytometry assay

Cells were stained with the Annexin V/FITC Apoptosis Detection Kit I (BD Pharmingen, Franklin Lakes, NJ, USA) 48 h after receiving a 10 Gy radiation dose to look for signs of apoptosis. Using a Coulter-XL flow cytometer (Beckman Coulter Inc., Brea, CA, USA) and an EXPO32 ADC, we were able to capture and analyze pictures of apoptosis. The total apoptosis rate of tumor cells was calculated by adding the apoptosis rates observed in the early (lower-right) and late (upper-right). After 24 h, cells that had been irradiated with 10 Gy were collected and preserved in cold ethanol (1 mL PBS + 2 mL absolute ethanol) for studying the cell cycle. The cells were stained with a PI/RNase staining solution (BD Pharmingen; Franklin Lakes, NJ, USA). After that, we analyzed the cells cycles distributions (G1 and G2 phase) using flow cytometry (Coulter-XL; Beckman Coulter Inc., Brea, CA, USA) and Mod Fit 3.0 (Verity Software House, Inc., USA).

### Colony formation assay

CD44( + )-OSCC cells (200 cells/well) were plated in 6-cm tissue culture plates and irradiated at 0, 4, and 8 Gy, respectively. After 2 weeks, the cells were fixed in 4% paraformaldehyde for 20 min, stained with crystal violet, and then washed in PBS. Survival rate (%) was calculated as (number of colonies/number of cells plated)_irradiated_/(number of colonies/number of cells plated)_non-irradiated_, where 50 cells was used as the cutoff for excluding colonies.

### Caspase-3 activity assay

The day following cell seeding in 6-well plates, the cells were irradiated (10 Gy; single treatment) when they reached 70–80% confluence. At 24 h post-irradiation, cells were analyzed using a Caspase-3 apoptosis detection kit (Beyotime, Shanghai, China). Ac-DEVD-pNA (2 mM) was added to the cell lysis detection solution. After incubation for 2 h at 37 °C, absorbances (at 405 nm) were measured using an Epoch^TM^ spectrophotometer (BIO-TEK instruments Inc., Vermont, USA).

### Immunofluorescence assay

The experiment was conducted as previously described [13]. Briefly, we first prepared and dewaxed the paraffin-embedded tumor samples. Subsequently, the samples were rehydrated, followed by antigen retrieval and blocking. Cells were processed by permeabilization using Triton-X100 (1%) for 30 min and blocking in BSA (1%) for 30 min after fixing in paraformaldehyde (4%) at room temperature for 20 min. Then, overnight incubation at 4 °C with the primary antibodies β-catenin (1:50, GTX101435, GeneTex), SALL4 (1:50, GTX109983, GeneTex), CD44 (1:200, Abcam, ab254530), METTL3 (1:100, Proteintech Group, 15073-1-AP, 67733-1-Ig). The samples were then washed in PBS and incubated with fluorescent rabbit/mouse secondary antibody (1:100, Abbkine) at room temperature for 1 h. Fluorescent pictures were taken via the FluoView FV1000, a confocal laser scanning microscope (Olympus, Tokyo, Japan) after the nuclei were stained with an anti-fluorescence quencher containing DAPI for 5 min at room temperature.

### In vivo tumorigenicity assay

At the Laboratory Animal Center of the Fourth Military Medical University in Xi’an, China, we bought female BALB/c nude mice aged 4–6 weeks. Mice were injected subcutaneously in the left forelimb with a solution containing 1 × 10^7^ stably transfected CAL27-CD44(+) cells in 0.2 mL. After the tumor reached a volume of 100 mm^3^, the mice (Vector and SALL4 groups) were randomized into two groups: those who received 10 Gy of radiation (5 Gy, 2 times) and those that did not. Eventually, nude mice that develop a tumor of the anticipated size were split into 4 groups (*n* = 3 in each) and randomly allocated to receive either a vector injection, SALL4 overexpression, vector injection plus irradiation, or SALL4 overexpression injection plus irradiation. The volume of the tumor was measured using a Vernier caliper every two days (length × width^2^ × 1/2). Over the course of two weeks, the tumor’s development was tracked weekly. The Research at the Fourth Military Medical University and the Committee on the Use of Live Animals in Teaching and authorized all procedures using mice.

### Statistical analysis

GraphPad Prism 8.0 (GraphPad Software, California, USA) was employed for all the statistical work. There were at least three sets of each experiment done. The student’s unpaired t-test was employed to examine the correlation between the two groups. One-way ANOVA with Dunnett’s multiple comparisons was utilized instead for analyzing data from more than three groups. Means and standard deviations are approached for summarizing the data. Statistical significance was defined as a *P*-value less than 0.05 (*p < 0.05, **p < 0.01, ***p < 0.001).

## Results

### METTL3 is highly expressed in OSCC and is correlated with poor OSCC survival and radioresistance

**Fig. 1:**
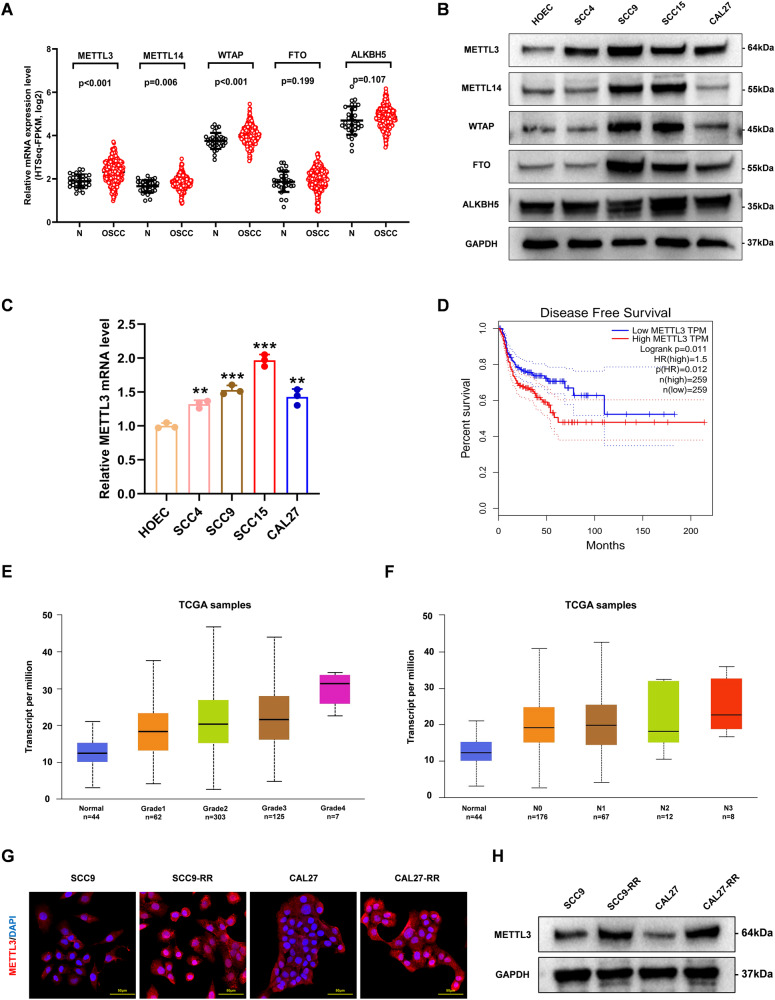
METTL3 is highly expressed in OSCC and is correlated with poor OSCC survival and radioresistance. **A** Results based on the TCGA databases showed the expression level of methyltransferase (METTL3, METTL14, WTAP), demethyltransferase (ALKBH5, FTO). **B** Western blotting analysis showed the protein levels of METTL3, METTL14, WTAP, ALKBH5, FTO in OSCC cell lines (SCC4, SCC9, SCC15, CAL27) and HOEC. **C** qRT-PCR analysis of METTL3 in OSCC cells. **D** Disease-free survival rate analysis based on the GEPIA platform (http://gepia.cancer-pku.cn/). **E** METTL3 expression levels based on tumor grade. **F** METTL3 expression levels based on nodal metastasis status. **G**, **H** Immunofluorescence images and western blot showing the METTL3 protein expression level in radioresistant and parental cells.

To demonstrate the role of m6A modifications on OSCC tumorigenesis, major m6A modification-related enzyme genes in 32 normal and 330 OSCC tissues based on the Cancer Genome Atlas database (TCGA: https://portal.gdc.cancer.gov/), and expression was analyzed, we found upregulation of methyltransferases expression (METTL3, METTL14, and WTAP) in OSCC tissues; however, demethylases FTO and ALKBH5 expression was insignificant (Fig. 1A). Furthermore, western blotting analysis revealed the elevated expression of METTL3 in all OSCC cell lines in comparison to that in normal oral epithelial cells (HOEC) (Fig. 1B). High expression of METTL3 was also observed in OSCC cell lines using RT-qPCR (Fig. 1C). In a cohort of patients with OSCC, high METTL3 expression was correlated with low disease-free survival (Fig. 1D) and high tumor grade and lymph nodes metastasis (Fig. 1E, F), indicating that METTL3 overexpression is related to OSCC poor prognosis. Importantly, both immunofluorescence and western blotting showed that METTL3 expression was higher in radioresistant cells compared with parental cells (Fig. 1G, H), suggesting that high METTL3 expression may be associated with radioresistance in OSCC.

### METTL3 promotes CSCs properties of CD44( + )-OSCC cells

**Fig. 2:**
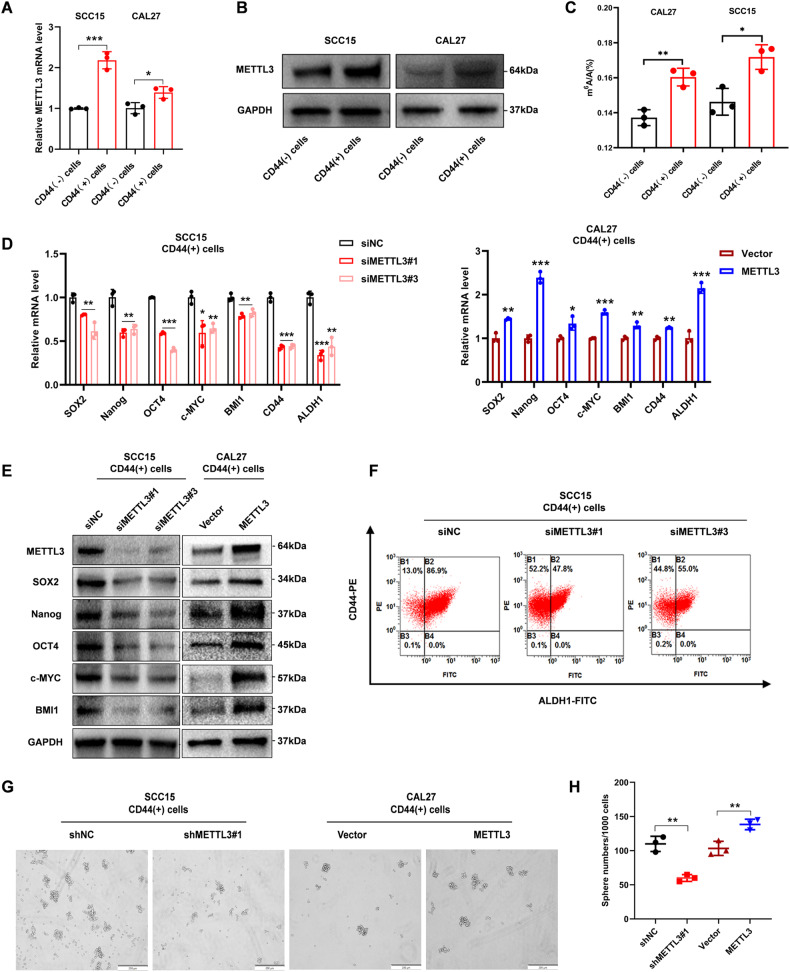
METTL3 promotes CSCs properties of CD44( + )-OSCC cells. **A** qRT-PCR analysis of METTL3 in CD44(+) cells and CD44(-) cells. **B** Western blotting analysis of METTL3 in CD44(+) cells and CD44(-) cells. **C** The global m6A modification level in CD44(+) cells and CD44(-) cells. **D** mRNA expression of CSCs-related genes. **E** Western blotting analysis of CSCs-related proteins. **F** Percentage of ALDH1/CD44 in negative controls and METTL3 knockdown as determined by flow cytometry. **G** Tumor sphere assay revealed that the upregulation of METTL3 elevated the CD44(+) cells sphere formation potential, while METTL3 knockdown diminished it. Scale bars: 200 μm. **H** Statistical analysis of data from the tumor sphere formation assay.

To explore the biological effects of METTL3 in OSCC CSCs. First, we sorted out CD44( + )-OSCC cells and CD44(-)-OSCC cells, by analyzing METTL3 expression in CD44 cells subpopulation, we observed that METTL3 was highly expressed in CD44( + )-OSCC cells (Fig. 2A, B). Consistently, METTL3 may be vital for OSCC CSCs since the global m6A modification level was considerably greater in CD44(+) cells than in CD44(-) cells (Fig. 2C). When we silenced METTL3 expression, the expressions of CSCs-related genes, like Nanog, SOX2, OCT4, c-MYC, and BMI1, along with the expressions of OSCC CSCs markers CD44 and ALDH1 were reduced. In contrast, overexpression of METTL3 upregulated the expression of CSCs-related genes (Fig. 2D, E). Flow cytometry analysis also showed a significantly reduced proportion of OSCC CSCs markers after METTL3 silencing (Fig. 2F). Tumor sphere assay conducted using cells with stably silenced (SCC15-CD44+) or overexpressed (CAL27-CD44+) METTL3 showed that METTL3 upregulation promoted CD44(+) self-renewal potential (Fig. 2G, H). These results suggested that METTL3 promotes the CSCs features of CD44( + )-OSCC cells.

### METTL3 promotes SALL4 mRNA translation and m6A modification lead to enhanced CSCs phenotype in CD44( + )-OSCC cells

**Fig. 3:**
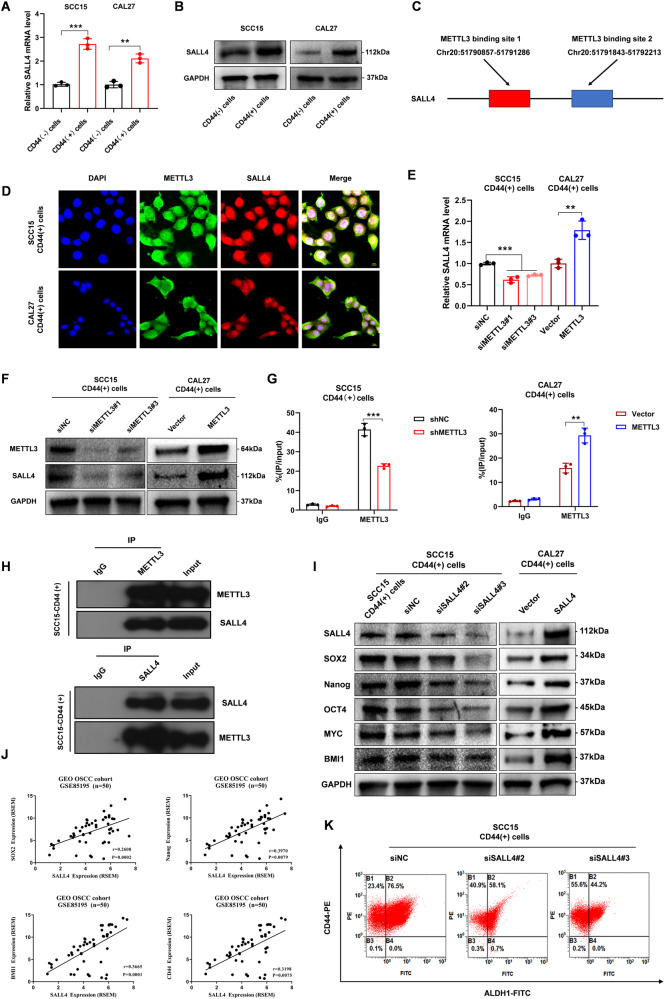
METTL3 promotes SALL4 mRNA translation and m6A modification lead to enhanced CSCs phenotype in CD44( + )-OSCC cells. **A**, **B** SALL4 mRNA and protein expression in CD44(+) cells and CD44(-) cells. **C** Predicted binding sites for SALL4 mRNA with METTL3. **D** The subcellular locations of METTL3 and SALL4, as detected by Immunofluorescence assay. Scale bars: 10 μm. **E**, **F** qRT-PCR and western blot analysis showed the SALL4 expression in CD44( + )-OSCC cells transfected with METTL3 silencing or overexpression. **G** RIP-qPCR showed the interaction between METTL3 and SALL4 mRNA. **H** The interaction of METTL3 and SALL4 was confirmed by CO-IP assay in CD44( + )-OSCC cells. IgG served as a negative control. **I** Relative protein expression levels of CSCs-related genes. **J** Positive correlation of SOX2, Nanog, BMI1, and CD44 with SALL4 as per the GEO-OSCC platform. **K** ALDH1/CD44 percentage in the negative controls, and the flow cytometry of cells with SALL4 knockdown.

To examine the mechanistic events of the regulation of OSCC CSCs by METTL3, we employed RNA sequencing to identify DEGs associated with the maintenance of CSCs phenotypes in parent and radioresistant cells (Supplementary Fig. 1A). Analysis of SALL4 mRNA and protein levels in OSCC cells revealed that SALL4 was highly expressed in OSCC (Supplementary Fig. 1B, C). These results were consistent with those observed in the TCGA database (Supplementary Fig. 1D). Furthermore, SALL4 demonstrated remarkably elevated expression levels in CD44( + )-OSCC cells (Fig. 3A, B), indicating that SALL4 possibly has a role in regulating the CSCs phenotype in OSCC.

To further understand the interaction between METTL3 and SALL4, relevant sequencing and prediction data for SALL4, the target gene of METTL3, were retrieved from the RM2Target database (http://rm2target.canceromics.org/#/home). The results indicate that there are two METTL3-mediated m6A sites at bases 383 to 758 and 1319 to 1755 of the SALL4 transcript (NM_020436) (Fig. 3C). Importantly, immunofluorescence experiments demonstrated the subcellular location of METTL3 and SALL4 in CD44( + )-OSCC cells, and mainly expressed in the nucleus (Fig. 3D). We employed METTL3 silenced and overexpression and found that the silencing and overexpression of METTL3 decreased and increased mRNA and protein levels of SALL4, respectively (Fig. 3E, F). Consistently, the GEPIA database also showed that METTL3 has a positive correlation with SALL4 expression in OSCC (Supplementary Fig. 1E). Using RNA immunoprecipitation-qPCR, we also found that METTL3 interacted directly with the SALL4 mRNA, and the m6A modification level of SALL4 decreased and increased after METTL3 silencing and overexpression, respectively (Fig. 3G). Subsequently, immunoprecipitation assays further confirmed that the direct binding of METTL3 to SALL4 (Fig. 3H). Furthermore, we also observed reduced and enhanced expression of CSCs-related genes after SALL4 knockdown and overexpression, respectively (Fig. 3I, Supplementary Fig. 1F). When we analyzed data from 50 OSCC tissues in the GEO database, SALL4 had a positive correlation with the expression of SOX2, Nanog, BMI1, and CD44 (Fig. 3J). SALL4 knockdown also reduced the proportion of OSCC CSCs markers CD44(+) and ALDH1(+) (Fig. 3K). Hence, these results suggested that METTL3 promotes SALL4 expression, leading to CSCs phenotype in CD44( + )-OSCC cells.

### SALL4 boosts the radioresistance of CD44( + )-OSCC cells via the Wnt/β-catenin signaling pathway

**Fig. 4:**
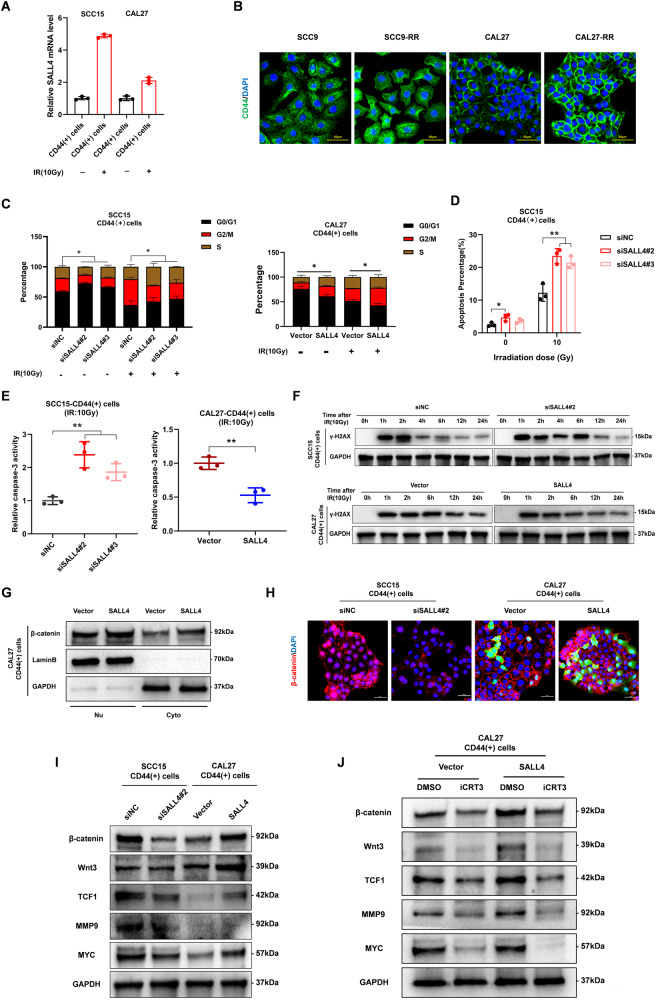
SALL4 boosts the radioresistance of CD44( + )-OSCC cells via the Wnt/β-catenin signaling pathway. **A** mRNA levels of SALL4 expression in CD44( + )-OSCC cells after irradiation. **B** Representative immunofluorescence images showing the CD44 protein expression level in radioresistant and parental cells. Scale bars: 50 μm. **C** Flow cytometry assessment of cellular cycles. SALL4 knockdown and overexpression diminished and increased cells in the G2/M phase, respectively. **D** SCC15-CD44(+) cells apoptosis was detected via flow cytometry. **E** Relative Caspase-3 activity of CD44( + )-OSCC cells, with knockdown or overexpression of SALL4, 24 h after irradiation at 10 Gy. **F** CD44( + )-OSCC cells were irradiated at 10 Gy and recultured under suitable circumstances for 1, 2, 4, 6, 12, and 24 h. Western blotting showing the levels of γ-H2AX. 0 h, irradiated cells without DNA repair time. **G** Expression of β-catenin in the cytoplasm and nucleus after SALL4 overexpression. **H** The nuclear localization and expression level of β-catenin in CD44( + )-OSCC cells from the indicated groups detected by immunofluorescence staining. (red fluorescence indicates β-catenin, and green fluorescence indicates zsgreen). Scale bars: 50 μm. **I** Western blotting analysis showing β-catenin, Wnt3a, TCF1, c-MYC, and MMP9 expression in CD44( + )-OSCC cells after SALL4 knockdown and overexpression, and **J** following treatment with iCRT3.

Several studies in the past have highlighted CSCs resistance to RT [16, 17]. And we found a critical biological influence of SALL4 on CD44( + )-OSCC cells. For additional evaluation of SALL4 role in regulating radioresistance in OSCC CSCs, we irradiated SCC15-CD44(+) and CAL27-CD44(+) cells and demonstrated a remarkable elevation in the expression levels of SALL4 mRNA and protein following irradiation (Fig. 4A, Supplementary Fig. 2A). We also detected a high level of SALL4 expression in radioresistance cells more than parent ones (Supplementary Figs. 2B–D). In addition, CD44, a tumor stem cell marker for oral squamous cell carcinoma, showed high expression in radioresistant cells (Fig. 4B, Supplementary Fig. 2E), indicating the possibility that SALL4 is associated with radioresistance in CD44( + )-OSCC cells. Cell cycle comparisons before and after radiation treatment indicated that SALL4 knockdown remarkably diminished the cellular population in the G2/M phase; however, SALL4 overexpression elevated it (Fig. 4C, Supplementary Fig. 2F), indicating that overexpression of SALL4 enhanced both DNA repair and radiotherapy resistance. Consistently, clonogenic assays also revealed decreased and increased radioresistance ability of CD44(+) cells when SALL4 was knocked down and overexpressed, respectively (Supplementary Fig. 2G, H). We also found that SALL4 knockdown enhanced the rate of apoptosis of CD44(+) cells after irradiation (Fig. 4D, Supplementary Fig. 2I). Furthermore, the overexpression of SALL4 significantly decreased Caspase-3 activity and enhanced anti-apoptotic ability after irradiation, whereas SALL4 knockdown remarkably elevated Caspase-3 activities following irradiation (Fig. 4E). The γ-H2AX histone is an important indicator of DNA damage, western blot showed that SALL4 overexpression additionally elevated the efficiency of DNA damage repair following irradiation, and vice versa for knockdown (Fig. 4F). In conclusion, SALL4 promotes the radioresistance of CD44( + )-OSCC cells.

The signaling pathway of Wnt/β-catenin is essential for the maintenance of features and radioresistance in a variety of CSCs [18]. We found that SALL4 overexpression promoted nuclear translocation of β-catenin, which was inhibited with SALL4 knockdown (Fig. 4G, H, Supplementary Fig. 2J). Further, SALL4 overexpression promoted the upregulation of Wnt3a and β-catenin expression and activated the downstream targeted genes (like TCF1, MYC, and MMP9) expression, when SALL4 was silenced, opposite changes were observed (Fig. 4I). When we used a Wnt/β-catenin pathway inhibitor (iCRT3), the results showed iCRT3 significantly abolished downstream target genes, β-catenin, and Wnt3a expression (Fig. 4J). These results suggest that SALL4 promotes radioresistance in CD44( + )-OSCC cells through the Wnt/β-catenin signaling pathway.

### METTL3/SALL4 promotes CSCs features and radioresistance in CD44( + )-OSCC cells

**Fig. 5:**
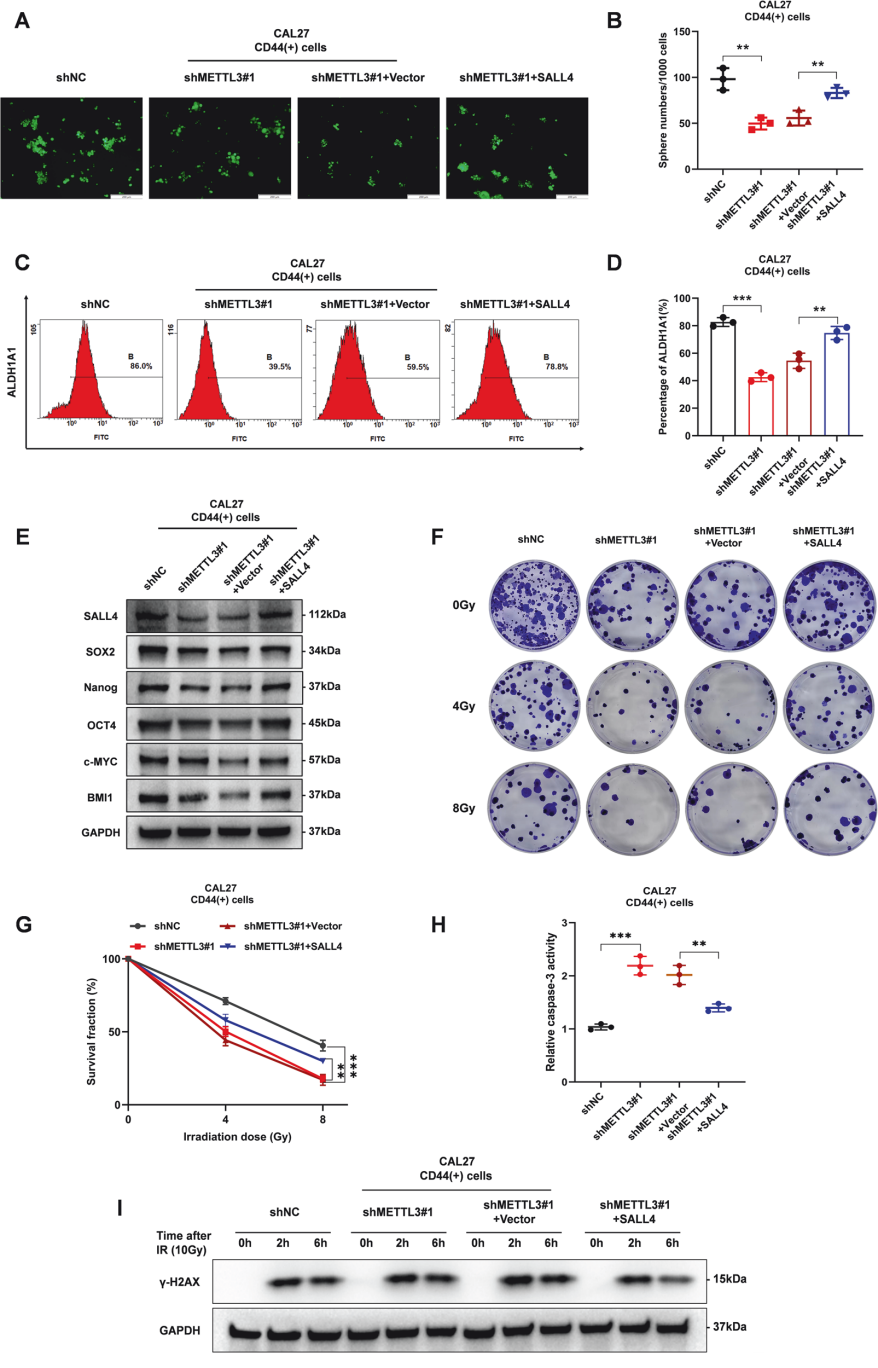
METTL3/SALL4 promotes CSCs features and radioresistance in CD44( + )-OSCC cells. **A** METTL3 knockdown inhibited sphere formation potential, which was partially restored by SALL4 overexpression (*n* = 3 independent experiments). Scale bars: 200 μm. **B** Statistics of the tumor sphere formation assay. **C** ALDH1A1 percentages in the negative control, shMETTL3, shMETTL3+vector, and shMETTL3 + SALL4 groups as flow cytometry revealed. **D** ALDH1A1 proportion statistic data in each group. **E** Western blot analysis showed the protein levels of CSCs-related factors in the relevant groups. **F** Representative images of clonogenic assay. **G** Clonogenic survival fraction were performed using CAL27-CD44(+) cells in the relevant groups at radiation doses of 0, 4, and 8 Gy. **H** Relative Caspase-3 activity 24 h following irradiation (10 Gy) of CAL27-CD44(+) cells in the indicated groups. **I** Levels of γ-H2AX in CAL27-CD44(+) cells irradiated at 10 Gy and recultured under suitable circumstances for 0, 2, and 6 h.

We constructed CAL27-CD44(+) cells with stable METTL3 knockdown and SALL4 overexpression simultaneously. Tumor sphere assays evaluation revealed the remarkable reduction the sphere-forming ability of CD44( + )-OSCC cells following METTL3 knockdown, which could be partially reversed it by SALL4 overexpression (Fig. 5A, B). Flow cytometry analysis the proportion of ALDH1A1, which is a marker of OSCC CSCs, decreased during METTL3 silencing and increased when SALL4 was overexpressed (Fig. 5C, D). Furthermore, METTL3 silencing suppressed the expression of CSCs-related genes; this effect was reversed during SALL4 overexpression (Fig. 5E). Using the clone formation and Caspase-3 activity assays, we further demonstrated that SALL4 reversed the altered radioresistance phenotype induced by METTL3 knockdown (Fig. 5F, G, H). This effect was additionally verified by γ-H2AX levels and DNA repair efficiency following irradiation (Fig. 5I). Taken together, our results demonstrate that METTL3-mediated transcriptional regulation of SALL4 promotes CSCs features and radioresistance in CD44( + )-OSCC cells.

### SALL4 promotes therapeutic resistance of OSCC tumor in vivo

**Fig. 6:**
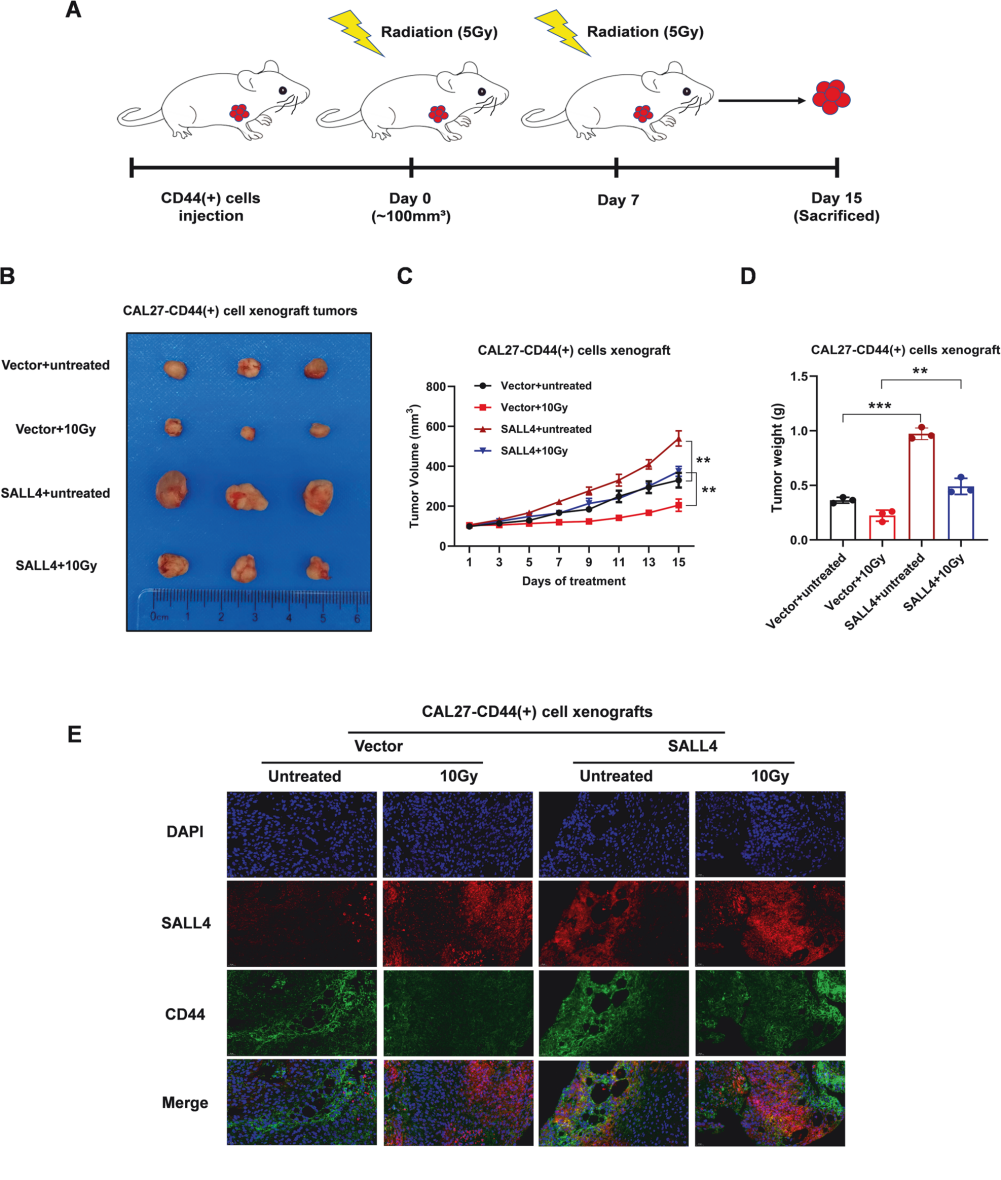
SALL4 promotes therapeutic resistance in OSCC tumors in vivo. **A** CAL27-CD44(+) cell line with a stable transfection subcutaneously injected into mice, followed by radiation therapy. **B** Representative tumor images from treated and untreated groups on the 15th day. **C** Growth curvatures of CAL27-CD44(+) cell xenografts. **D** Tumor weight of CAL27-CD44(+) cell xenografts. **E** Immunofluorescence analysis of treated tumors for CD44 (green) and SALL4 (red) expression. Scale bar = 20 µm.

The stable transfection CAL27-CD44(+) cells were subcutaneously inoculated into the left forelimbs of study animals. After the tumor reached a volume of 100 mm^3^, the mice (Vector and SALL4 groups) were randomized into two groups: those who received 10 Gy of radiation (in two doses, 5 Gy/fraction) and those that did not. For a total of 2 weeks, every two days measurements of the tumor were taken (Fig. 6A). Inhibitory therapeutic effects of SALL4 overexpression were clearly visible in comparison to the control group (Fig. 6B). Compared to the SALL4 + IR group, tumor volume and weight were drastically lower in the vector + IR group (Fig. 6C, D). Following SALL4 overexpression, there was an increase in the expression of the CSCs marker CD44 in OSCC. The SALL4 overexpression group also showed elevated CD44 expression following treatment (10 Gy) more than controls (Fig. 6E). These findings supported the hypothesis that SALL4 has a role in promoting RT resistance in OSCC in vivo.

## Discussion

OSCC is a malignant tumor with a poor prognosis, accounting for more than 90% of all oral cancers. Radiotherapy is a common treatment for patients with OSCC; the treatment effectively inhibits tumor growth, increases the probability of patient survival, and improves patients’ quality of life [19]. However, the response of patients with OSCC varies widely, with increasing incidence of resistance to RT [20], greatly limiting the RT efficacy. Hence, improving the sensitivity of patients with OSCC to RT and reversing radioresistance has become a major clinical challenge in this field.

Recent studies have established a close association of CSCs with RT resistance. For example, in head and neck tumors, the ERK1/2-Nanog signaling pathway promotes CSCs phenotyping together with epithelial-mesenchymal transition process, enhancing RT resistance and metastasis [21]. Furthermore, another study found that during RT, the JAK2/STAT3 signaling pathway was activated, which in turn activated downstream CCND2 expression and enhanced CSCs properties, leading to radioresistance in colorectal cancer [16]. Targeting CSCs and signaling pathway proteins in several kinds of tumors helped overcome radioresistance and improve patient survival [22]. CD44 is a single-chain transmembrane glycoprotein and is considered a surface-specific marker of HNSCC CSCs [23]. CD44(+) cells with CSCs features are correlated with HNSCC poor prognosis [24]. Using in vivo experiments, it has also been shown that CD44(+) cells have a more elevated tumor initiation potential than the CD44(-) ones [25]. High CD44 expression reflects the aggressiveness of tumor cells and the malignancy of HNSCC [26]. In the present study, we obtained subpopulations of CD44(+) cells using an immunomagnetic bead sorting method and found that CD44(+) cells had significantly higher ability of self-renewal, apoptosis inhibition, and clone formation compared with CD44(-) cells. Hence, CD44 can be used as an effective molecular surface marker for the enrichment of OSCC CSCs.

m6A modification has been frequently associated with several tumors; the modification process is regulated mainly by methyltransferases, demethylases, and methylation recognition enzymes [9]. Although previous studies have well-established the function of METTL3 in human cancers, including OSCC [8, 27], whether METTL3 has a regulatory role in OSCC CSCs still needs further elucidation. We observed that METTL3 was expressed in a higher proportion of the CD44(+) cell subpopulation compared with the subpopulation of CD44(-) cells. We also showed high levels of m6A modification in CD44(+) cells, suggesting that this modification is possibly related to OSCC CSCs regulation. We additionally revealed that METTL3 overexpression promotes CSCs stemness maintenance. Our data are consisted to that reported for other tumors. For example, the METTL3-AFF4-SOX2/MYC novel signaling axis was shown to promote self-renewal and tumor heterogeneity in bladder cancer stem cells (BCSCs), demonstrating the importance of m6A modifications [28]. FZD10 activation in hepatocellular carcinoma CSCs was reported to be facilitated via METTL3-dependent m6A methylation; further, it was shown that FZD10 promotes self-renewal, tumorigenicity, and metastasis in hepatocellular carcinoma (HCC) CSCs via β-catenin and YAP1 activation, predicting a poor prognosis [29]. Furthermore, METTL3-mediated m6A modification was shown to promote stemness maintenance and radioresistance in glioma stem cells (GSCs), highlighting METTL3 as a promising therapeutic target in glioblastomas (GBMs) [30]. In the present study, several m6A modifying enzymes (especially METTL3, WTAP, and YTHDF1) were found to be upregulated in OSCC tissues in the TCGA database, indicating a connection between METTL3 and the OSCC development. Overall, the present study confirms that m6A modifications regulates OSCC CSCs and promotes the maintenance of stemness of CSCs.

A high expression of SALL4 is known in various tumors, such as breast cancer [31] and HCC [32]. Studies have demonstrated that SALL4 is related to aggressiveness and HNSCC worse prognosis [33]. Furthermore, targeting SALL4 is promising for treating OSCC [34]. Our results also reveal a role of SALL4 in OSCC onset and development. We found that SALL4 expression was upregulated in OSCC and promoted CSCs phenotype and radioresistance both in vitro and in vivo. These outcomes are similar to previous results. SALL4 is a potential target for the development of radiosensitizers, since it was shown in a study of nasopharyngeal carcinoma (NPC) to produce radioresistance through the ATM/Chk2/p53 pathway and its downstream apoptosis-relevant proteins [35]. In another study, SALL4 activation enhanced microsphere formation and invasion, which are key features of CSCs, and upregulation of SALL4 resulted in elevated expression of molecular markers KRT19, EPCAM, and CD44 in hepatic stem cells [36]. SALL4 expression is upregulated in EGFR-mutated lung cancer cells, which promoting lung cancer cells from invading and metastasizing [37]. Mechanistically, our results show that activation of the Wnt signaling pathway and subsequent radioresistance in OSCC are caused by an increase in SALL4 expression and the nuclear transport of β-catenin.

The Wnt/β-catenin signaling pathway has been remarkably related to many cancer aspects, like tumorigenesis, malignant progression, together with poor prognosis, and is considered to be the most attractive clinical target for disease treatment [38]. In addition to processes, such as β-catenin nuclear translocation and activation of target genes by TCF/LEF complex, other included components are wnt proteins, FZD, AXIN, LRP5/6, and GSK-3β [39]. Elevated expression of SALL4 following irradiation induced β-catenin nuclear translocation in our investigation, which is recognized by the TCF/LEF complex and activates transcription of Cyclin D1, TCF1, c-MYC, and MMP9. However, the addition of the Wnt inhibitor iCRT3 decreased SALL4 and downstream target genes and blocked β-catenin’s nuclear trafficking. But the mechanisms by which SALL4 translocates to the cytoplasm and activates β-catenin need to be explained. When Wnt signaling is low, however, β-catenin is destroyed, which in turn prevents the transcriptional activation of downstream genes [40]. The stimulation of the Wnt signaling pathway promotes carcinogenesis, thus future research should determine if SALL4 interacts with β-catenin directly or indirectly.

**Fig. 7:**
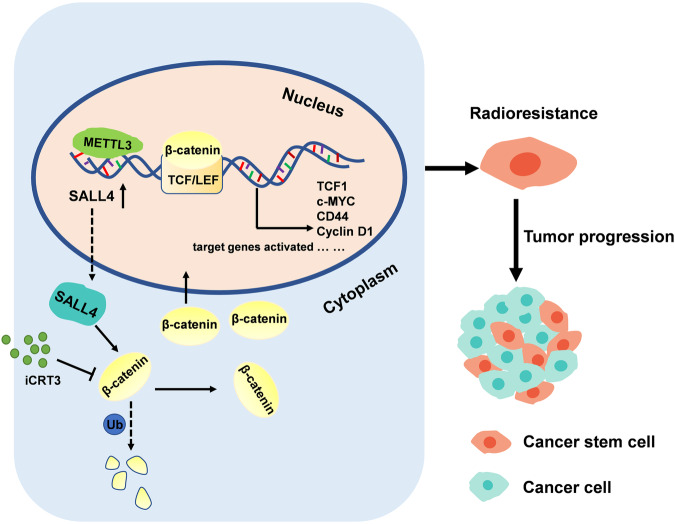
Schematic model depicting that SALL4 promotes OSCC radioresistance through Wnt/β-catenin signaling pathway.

In conclusion, this investigation showed that SALL4 is an important stem cell factor for causing resistance to RT in OSCC. Our data revealed that the METTL3/SALL4/Wnt/β-catenin axis promotes CSCs properties and radioresistance, ultimately facilitating the OSCC progression (Fig. 7). Outcome of this study may direct research toward utilizing newly-identified target for radiotherapy, malignancy progression, and prognosis prediction in OSCC.

### Supplementary information

Supplementary Figure1

Supplementary Figure2

Supplementary Figure3

supplementary material

Original Data File

## Data Availability

The datasets used or/and analyzed during the current study are available from the corresponding author on reasonable request.
