# Supplementary figures and images for: SALL4 promotes cancer stem-like cell phenotype and radioresistance in oral squamous cell carcinomas via methyltransferase-like 3-mediated m6A modification
Source: Cell Death Dis. 2024 Feb 14;15(2):139. doi: 10.1038/s41419-024-06533-9 (PMC10866932; doi:10.1038/s41419-024-06533-9)

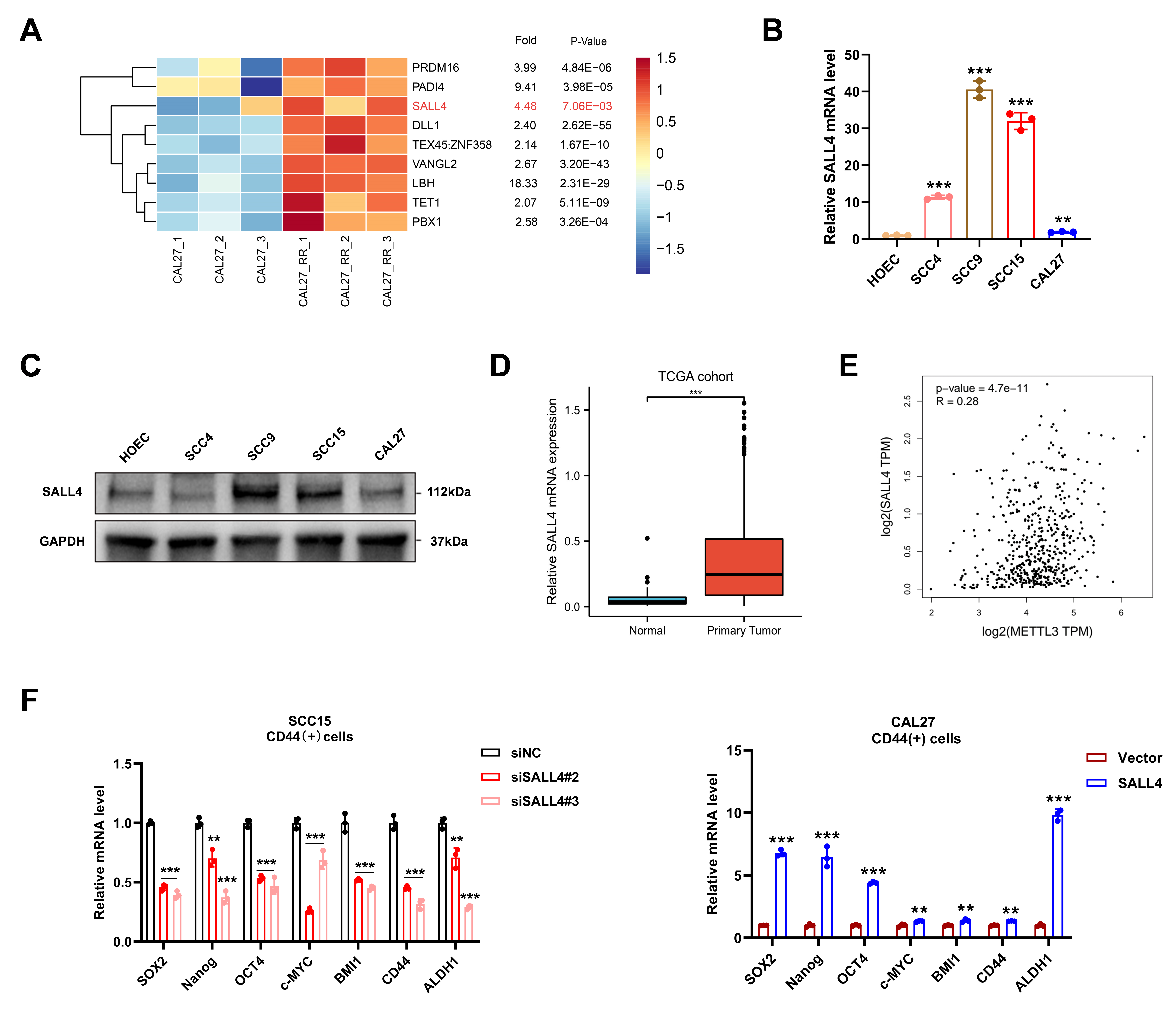

Supplement: Supplementary file 1 — Supplementary Figure1 [file 41419_2024_6533_MOESM1_ESM.tif]

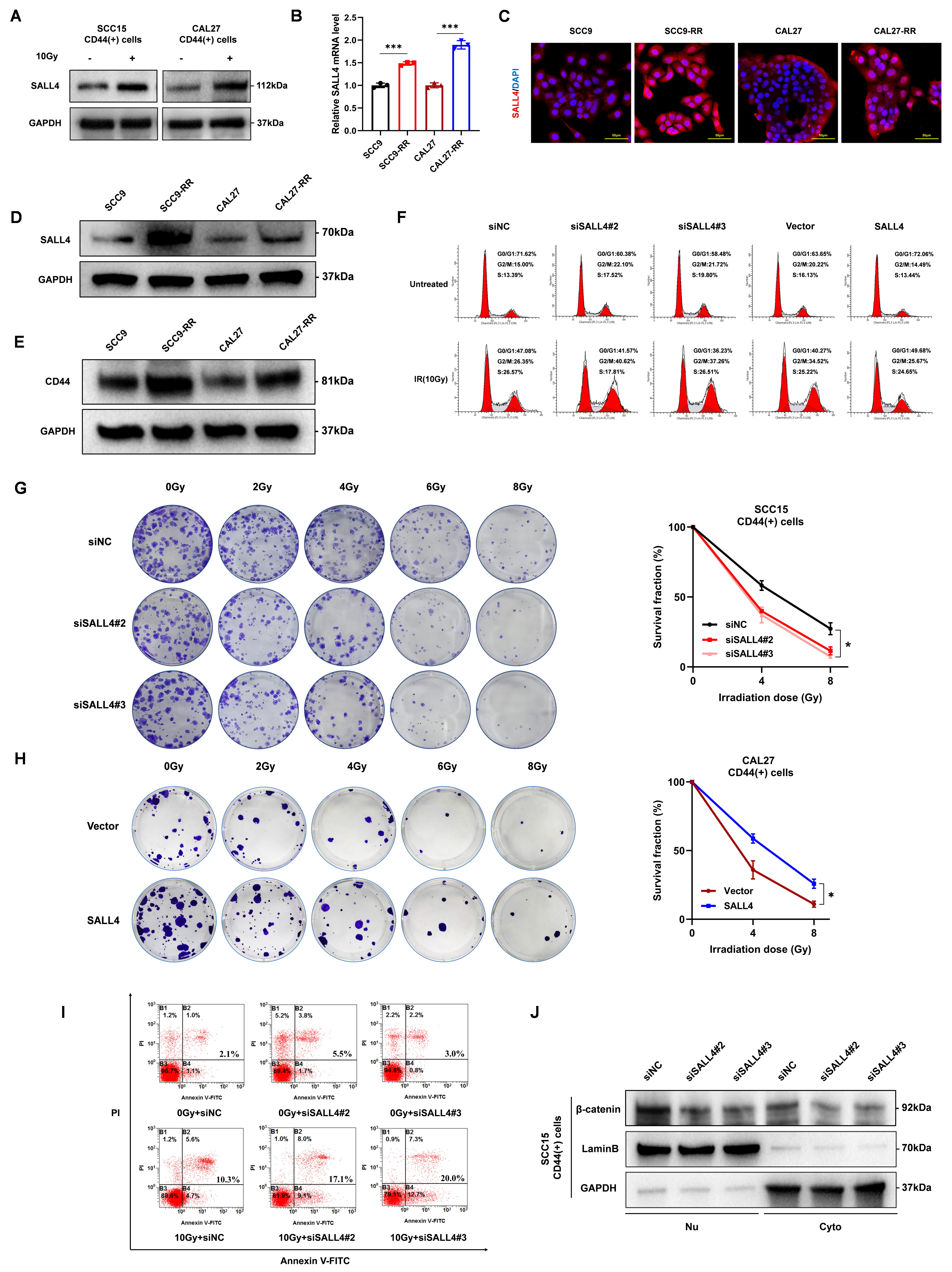

Supplement: Supplementary file 2 — Supplementary Figure2 [file 41419_2024_6533_MOESM2_ESM.tif]

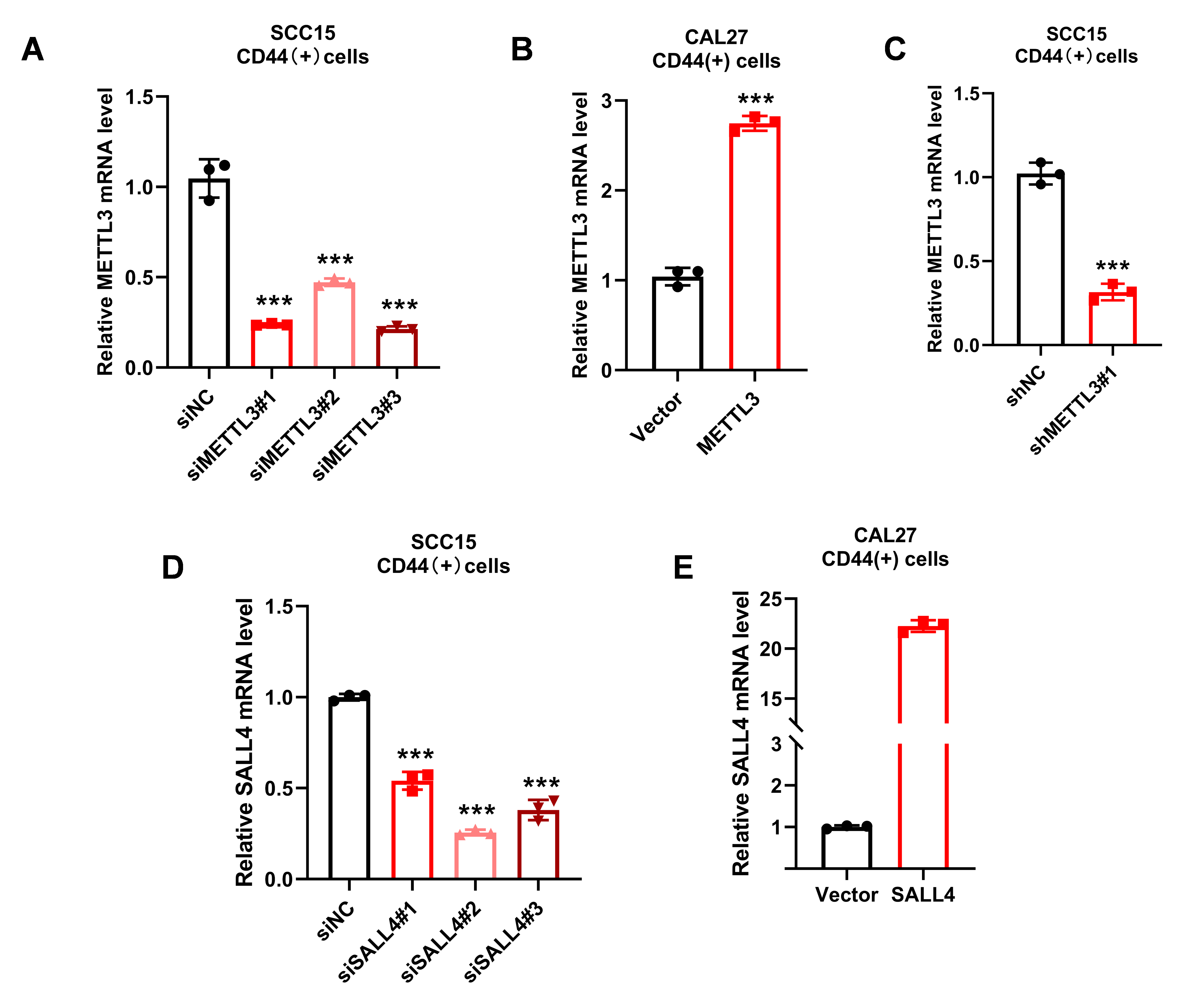

Supplement: Supplementary file 3 — Supplementary Figure3 [file 41419_2024_6533_MOESM3_ESM.tif]
