# Supplementary material for: SALL4 promotes cancer stem-like cell phenotype and radioresistance in oral squamous cell carcinomas via methyltransferase-like 3-mediated m6A modification
Source: Cell Death Dis. 2024 Feb 14;15(2):139. doi: 10.1038/s41419-024-06533-9 (PMC10866932; doi:10.1038/s41419-024-06533-9)
Supplement: Supplementary file 4 — supplementary material [file 41419_2024_6533_MOESM4_ESM.docx]

**Supplementary Figure 1.** (A) Screening of differentially expressed genes associated with stemness properties of CSCs from parent and radioresistant cells using RNA-seq. (B, C) mRNA and Protein expression of SALL4 in OSCC cell lines (CAL27, SCC15, SCC9, and SCC4) and HOEC. (D) SALL4 expression in OSCC tissues (n = 330) and normal (n =32) tissues from the TCGA database. (E) METTL3 positively correlated with SALL4 expression as assessed via GEPIA database ([gepia.cancer-pku.cn)](http://gepia.cancer-pku.cn/index.html). (F) qRT-PCR showed that expression levels of mRNA of CSCs-related genes after METTL3 silencing and overexpression.

**Supplementary Figure 2.** (A) Western blot analysis showed SALL4 protein levels after irradiation (10 Gy) of CD44(+)-OSCC cells. (B) SALL4 mRNA expression levels of radioresistant and parent cells. (C) Representative immunofluorescence images showing the SALL4 protein expression level in radioresistant and parental cells. Scale bars: 50 μm. (D, E) Western blot analysis indicated the SALL4 and CD44 protein expression level in radioresistant and parental cells. (F) The cell cycle proportion of CD44(+)-OSCC cells was detected by flow cytometry. (G, H) Representative images and statistical analysis of clonogenic assay in the indicated groups after irradiation (0-8Gy). (I) Apoptosis rate of SCC15-CD44(+) cells, as detected by flow cytometry. (J) β-catenin expression levels in the nucleus and cytoplasm after SALL4 knockdown in SCC15-CD44(+) cells.

**Supplementary Figure 3.** (A, B, C) METTL3 knockdown or overexpression effect in SCC15-CD44(+) cells or CAL27-CD44(+) cells transfected with METTL3 siRNA, overexpression plasmids, or shRNA. (D, E) SALL4 knockdown or overexpression effect in SCC15-CD44(+) cells or CAL27-CD44(+) cells transfected with SALL4 siRNA and overexpression plasmids.

**Supplementary Table1. The list of primer sequences**

| Primer |  | Sequence (5′−3′) |
| --- | --- | --- |
| METTL3 Forward |  | CTGTGTCCATCTGTCTTGCCATCTC |
| METTL3 Reverse |  | CCTCGCTTTACCTCAATCAACTCCTG |
| SALL4 Forward |  | GTTGGTGGAGAACATTGACAAG |
| SALL4 Reverse |  | CTTAATGGATGTGTTGGTTCGG |
| SOX2 Forward |  | CAGCATGTCCTACTCGCAGCAG |
| SOX2 Reverse |  | CTGGAGTGGGAGGAAGAGGTAACC |
| Nanog Forward |  | GATGCAAGAACTCTCCAACATC |
| Nanog Reverse |  | CTGGTGGTAGGAAGAGTAAAGG |
| OCT4 Forward |  | GTGGTCCGAGTGTGGTTCTGTAAC |
| OCT4 Reverse |  | CCCAGCAGCCTCAAAATCCTCTC |
| c-MYC Forward |  | CGACGAGACCTTCATCAAAAAC |
| c-MYC Reverse |  | CTTCTCTGAGACGAGCTTGG |
| BMI1 Forward |  | CAAGACCAGACCACTACTGAAT |
| BMI1 Reverse |  | TATCTTCATCTGCAACCTCTCC |
| CD44 Forward |  | TCTGAATCAGATGGACACTCAC |
| CD44 Reverse |  | CATTGCCACTGTTGATCACTAG |
| ALDH1 Forward |  | GACAATGCTGTTGAATTTGCAC |
| ALDH1 Reverse |  | AAGGATATACTTCTTAGCCCGC |
| GAPDH Forward |  | GCACCGTCAAGGCTGAGAAC |
| GAPDH Reverse |  | TGGTGAAGACGCCAGTGGA |

Primers for siRNA

| hMETTL3#1 sense  hMETTL3#1 antisense | GCCUUAACAUUGCCCACUGAUTT  AUCAGUGGGCAAUGUUAAGGCTT |
| --- | --- |
| hMETTL3#3 sense  hMETTL3#3 antisense | GCAAGUAUGUUCACUAUGAAATT  UUUCAUAGUGAACAUACUUGCTT |
| hMETTL3-NC sense  hMETTL3-NC antisense | UUCUCCGAACGUGUCACGUTT  ACGUGACACGUUCGGAGAATT |
| hSALL4-2# sense  hSALL4-2# antisense | CCAUAGAUGAACCGAGUCUUUTT  AAAGACUCGGUUCAUCUAUGGTT |
| hSALL4-3# sense  hSALL4-3# antisense | CCGACCUAUGUCAAGGUUGAATT  UUCAACCUUGACAUAGGUCGGTT |
| hSALL4-NC sense  hSALL4-NC antisense | UUCUCCGAACGUGUCACGUTT  ACGUGACACGUUCGGAGAATT |

**Supplementary Table2. Antibody information used in Immunofluorescence and western blot**

| Protein | Application | Origin | Dilution |
| --- | --- | --- | --- |
| SALL4 | WB&IF | GeneTex, GTX109983 | 1:500&1:50 |
| SALL4 | WB&IP | Proteintech Group, 24500-1-AP | 1:1000&1μg |
| METTL3 | WB | Abcam, ab195352 | 1:1000 |
| METTL3(Rabbit) | IF&IP | Proteintech Group, 15073-1-AP | 1:100&1μg |
| METTL3(Mouse) | IF | Proteintech Group, 67733-1-Ig | 1:100 |
| METTL14 | WB | Proteintech Group, 26158-1-AP | 1:1000 |
| WTAP | WB | Proteintech Group, 60188-1-Ig | 1:1000 |
| ALKBH5 | WB | Proteintech Group, 16837-1-AP | 1:1000 |
| FTO | WB | Proteintech Group, 27226-1-AP | 1:1000 |
| γ-H2AX | WB | Abcam, ab81299 | 1:1000 |
| SOX2 | WB | GeneTex,GTX627404 | 1:1000 |
| Nanog | WB | Abcam, ab109250 | 1:1000 |
| Oct4 | WB | Abcam, ab181557 | 1:1000 |
| c-Myc | WB | Abcam, ab32072 | 1:1000 |
| Bmi1 | WB | Abcam, ab126783 | 1:10000 |
| β-catenin | WB&IF | GeneTex, GTX101435 | 1:1000&1:100 |
| Wnt3a | WB | Abcam, ab28472 | 1:1000 |
| TCF1 | WB | Proteintech Group, 22426-1-AP | 1:1000 |
| MMP9 | WB | Abcam, ab283575 | 1:1000 |
| CD44 | IF | Proteintech Group, 15675-1-AP | 1:50 |
| CD44 | WB&IF | Abcam, ab254530 | 1:1000&1:200 |
| Lamin B | WB | Abcam, ab133741 | 1:1000 |
| GAPDH | WB | GeneTex, GTX637966 | 1:5000 |

Abbreviations: IF, Immunofluorescence; WB, western blot.
