# Supplementary material for: SALL4 promotes cancer stem-like cell phenotype and radioresistance in oral squamous cell carcinomas via methyltransferase-like 3-mediated m6A modification
Source: Cell Death Dis. 2024 Feb 14;15(2):139. doi: 10.1038/s41419-024-06533-9 (PMC10866932; doi:10.1038/s41419-024-06533-9)
Supplement: Supplementary file 5 — Original Data File [file 41419_2024_6533_MOESM5_ESM.pdf]

Figure1 B

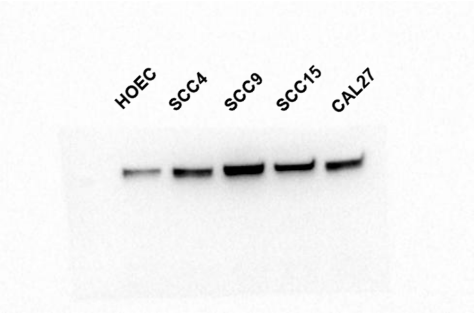

METTL3

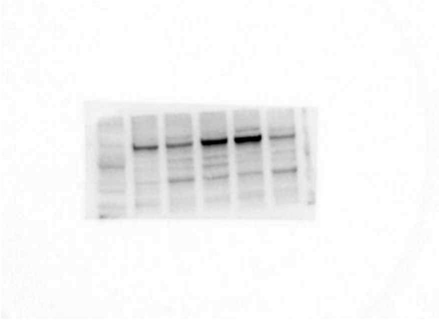

METTL14

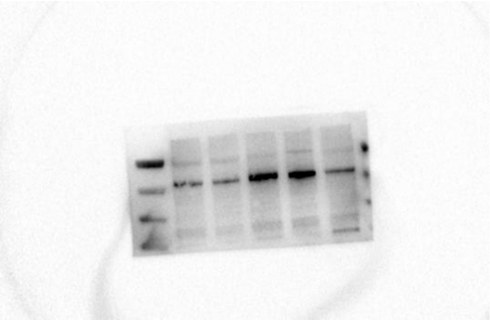

WTAP

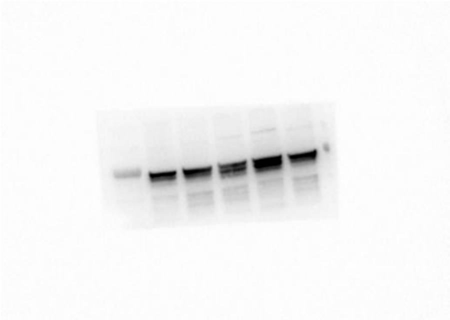

ALKBH5

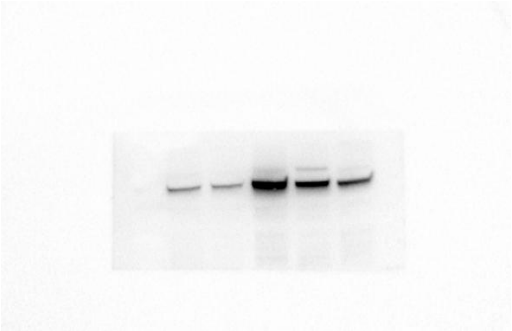

FTO

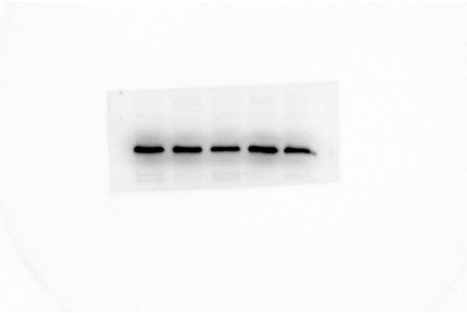

GAPDH

Figure1 H

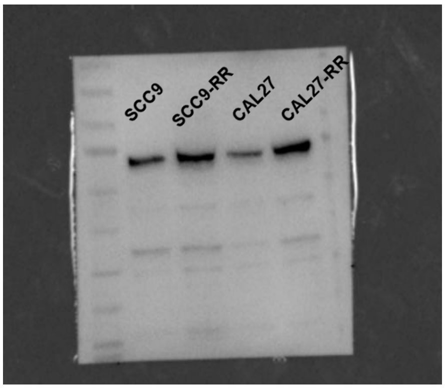

METTL3

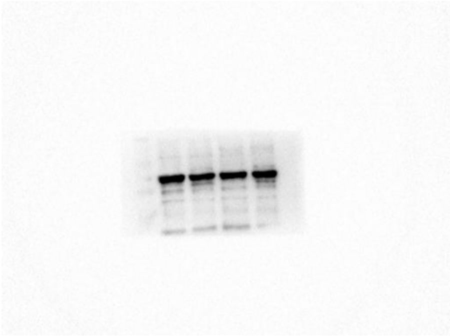

GAPDH

Figure2 B

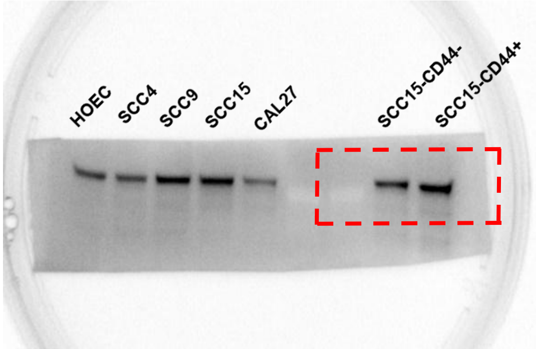

SCC15-METTL3

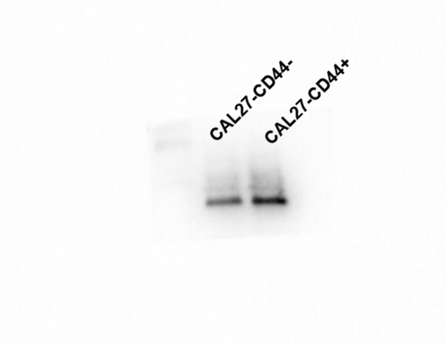

CAL27-METTL3

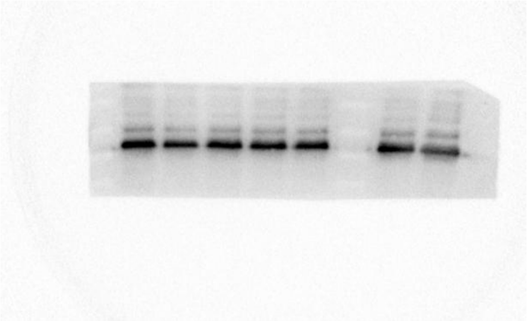

GAPDH

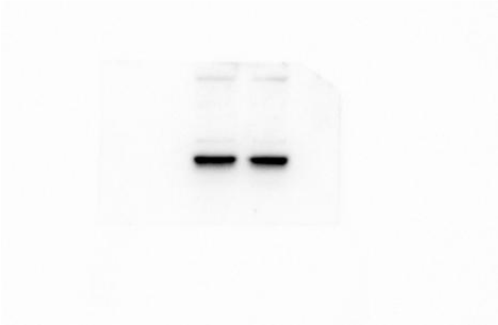

GAPDH

Figure2 E

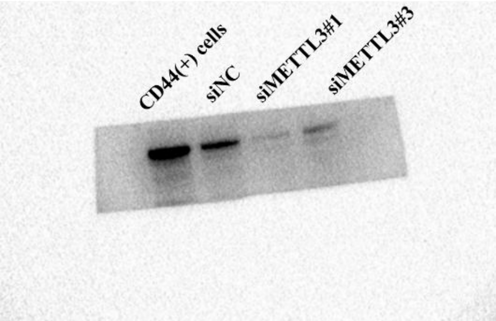

si-METTL3

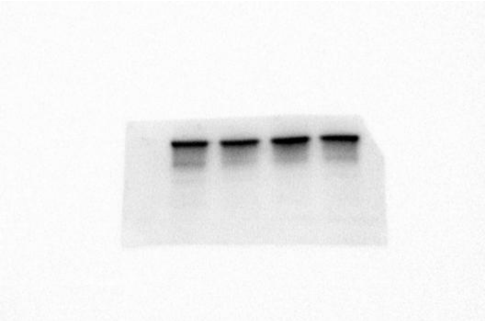

GAPDH

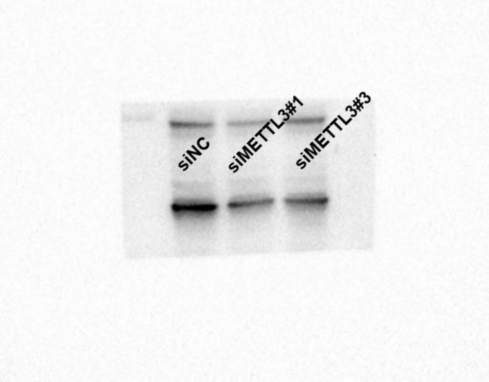

siMETTL3-SOX2

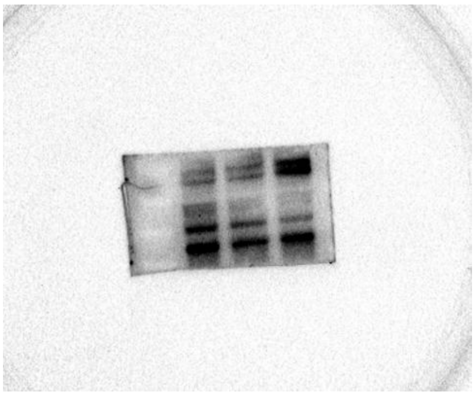

siMETTL3-Nanog

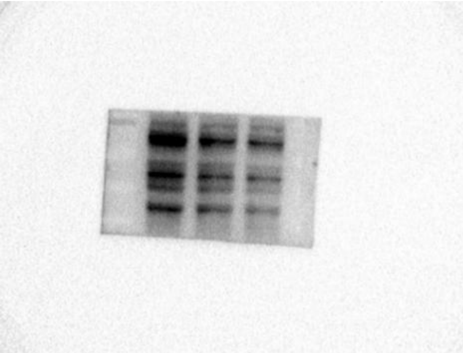

siMETTL3-OCT4

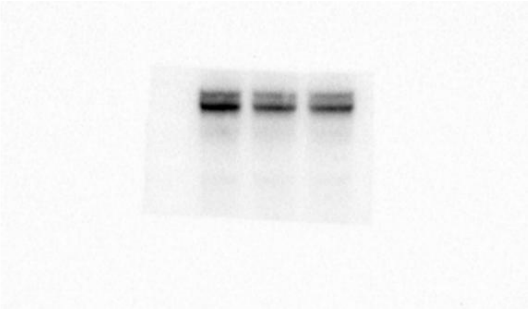

siMETTL3-c-MYC

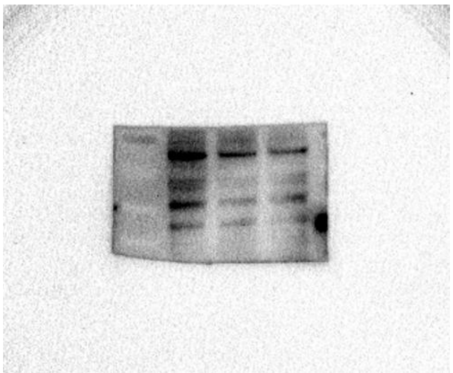

siMETTL3-BMI1

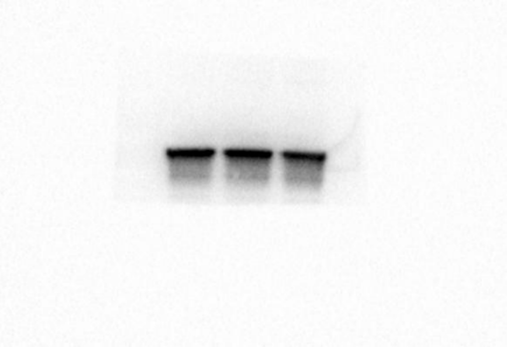

siMETTL3-GAPDH

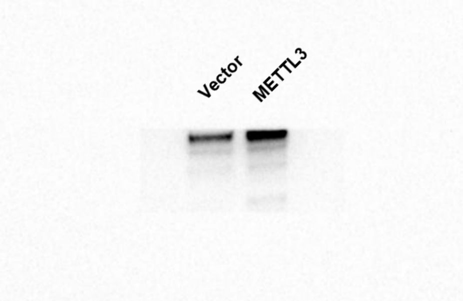

OE-METTL3

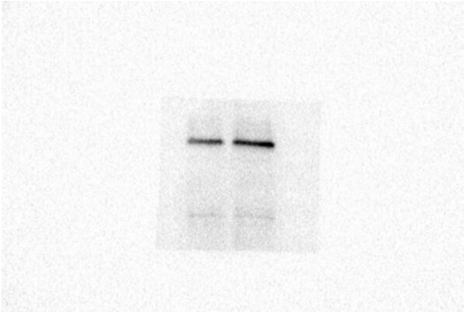

OE-METTL3-SOX2

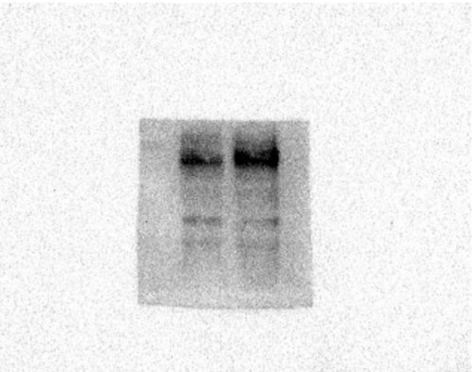

OE-METTL3-Nanog

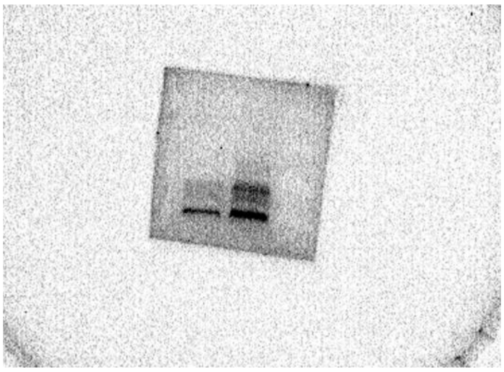

OE-METTL3-OCT4

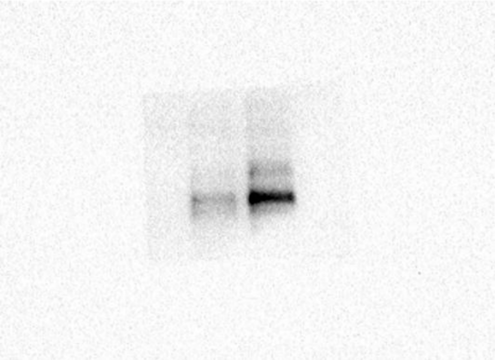

OE-METTL3-c-MYC

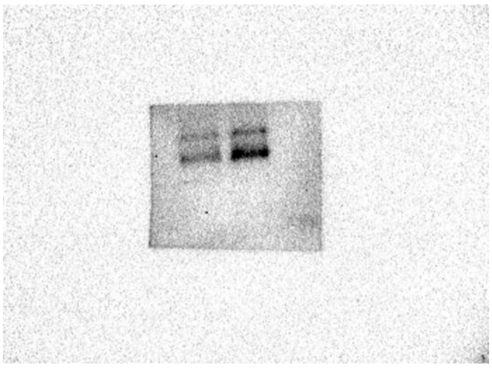

OE-METTL3-BMI1

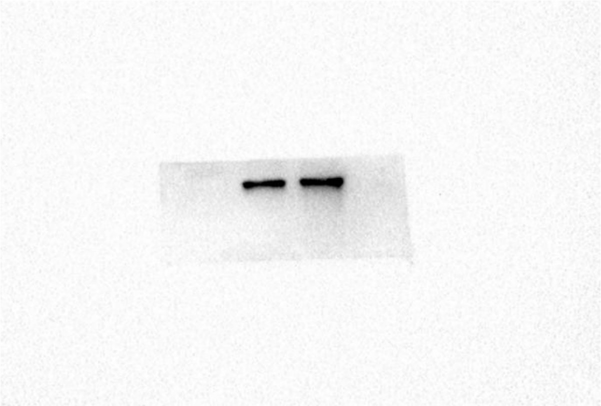

OE-METTL3-GAPDH

Figure3 B

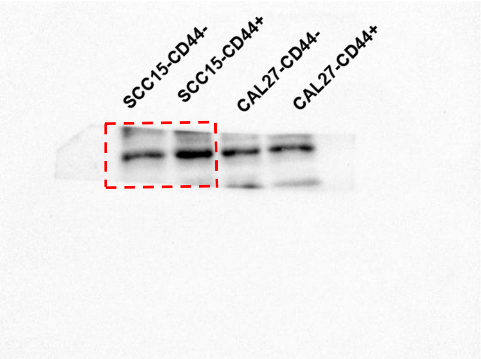

SCC15-SALL4

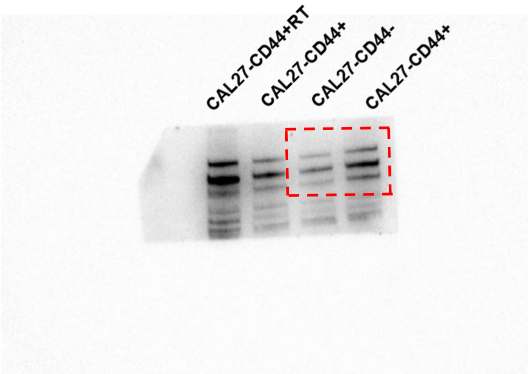

CAL27-SALL4

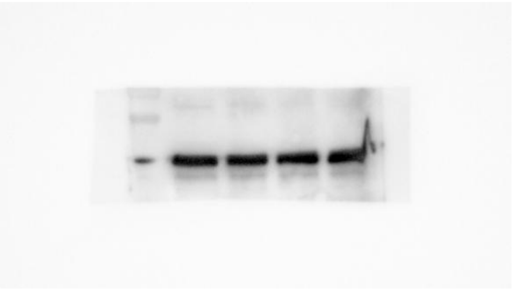

GAPDH

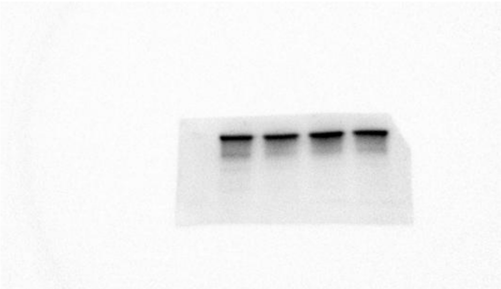

GAPDH

Figure3 F

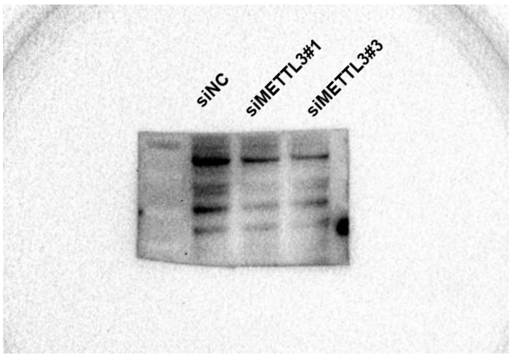

siMETTL3-SALL4

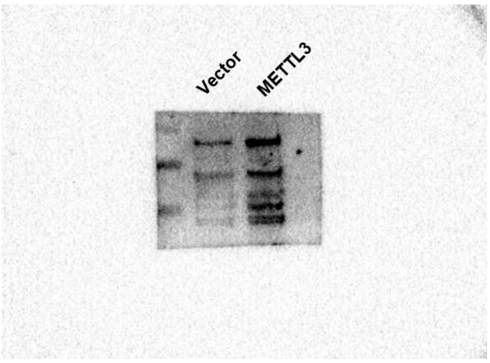

OE-METTL3-SALL4

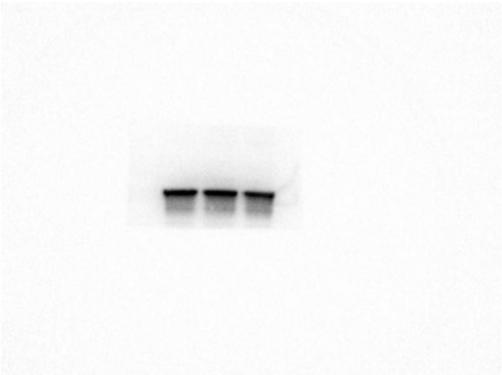

GAPDH

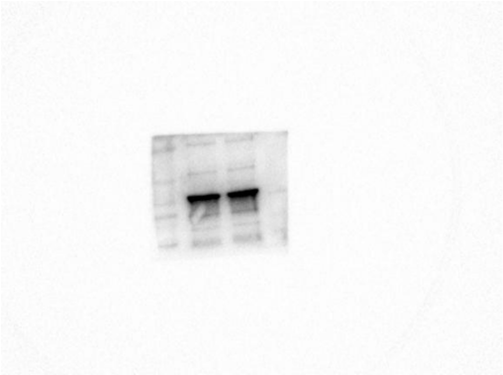

GAPDH

Figure3 H

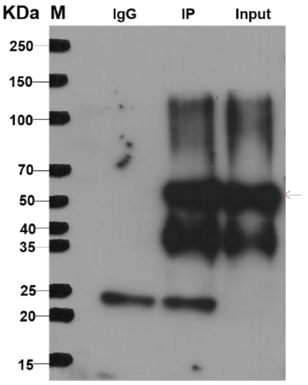

METTL3

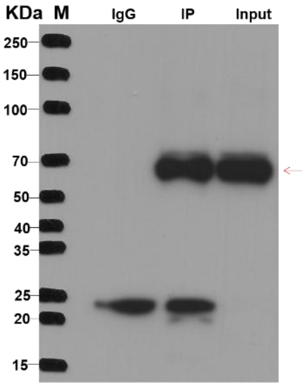

SALL4

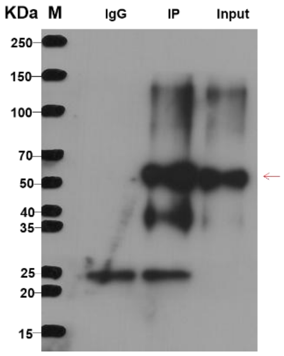

METTL3

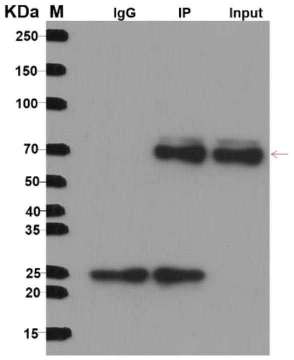

SALL4

Figure3 I

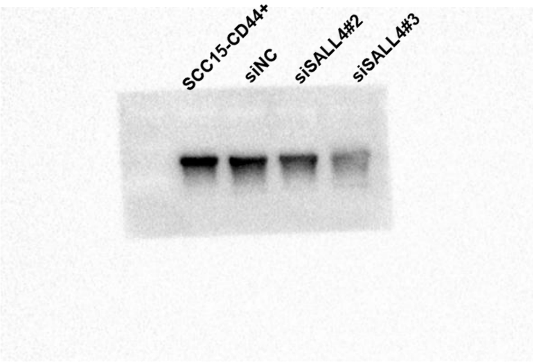

siSALL4-SOX2

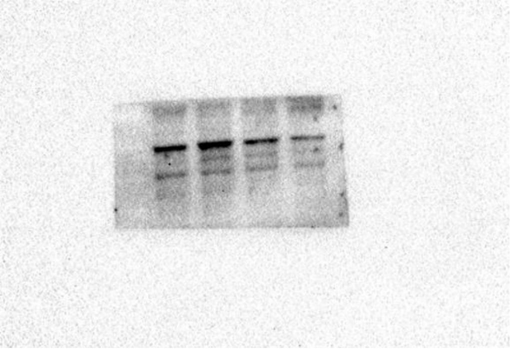

siSALL4-Nanog

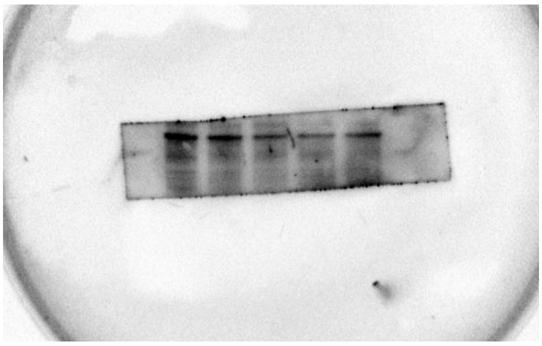

siSALL4-OCT4

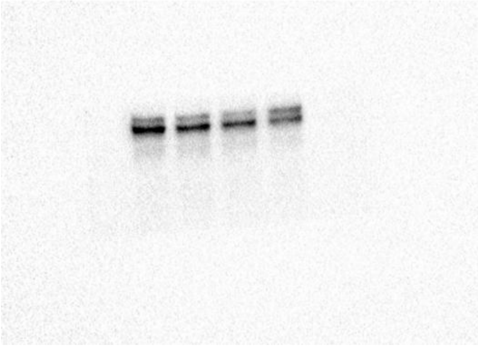

siSALL4-cMYC

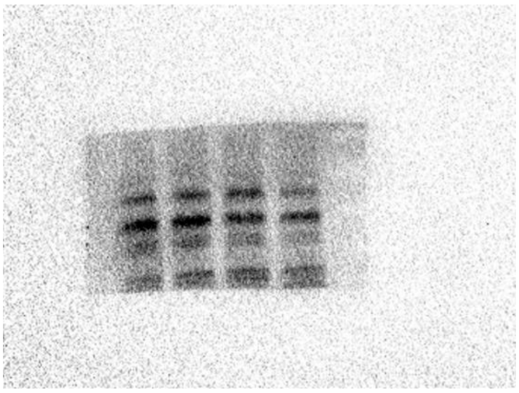

siSALL4-BMI1

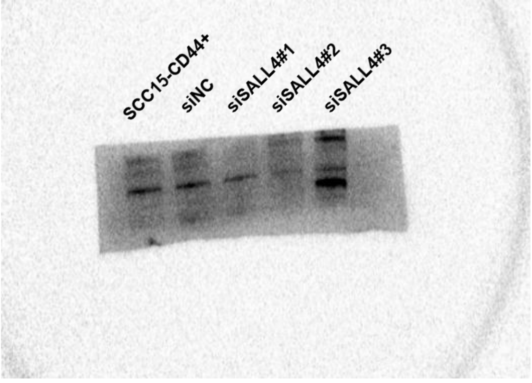

siSALL4

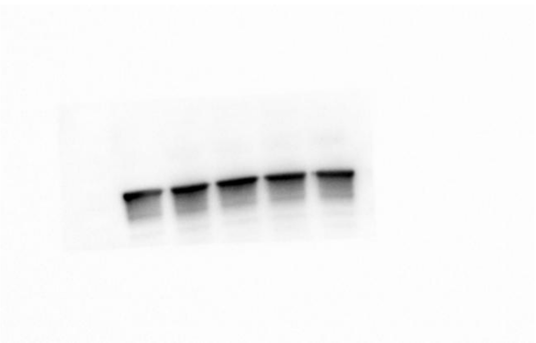

siSALL4-GAPDH

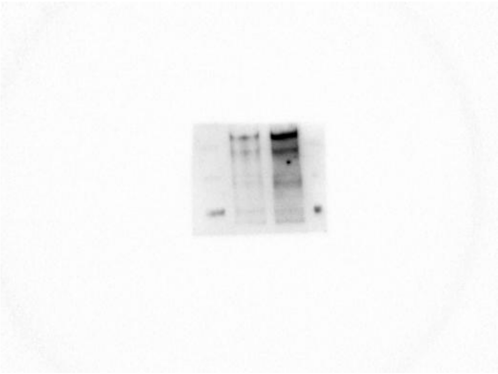

OE-SALL4

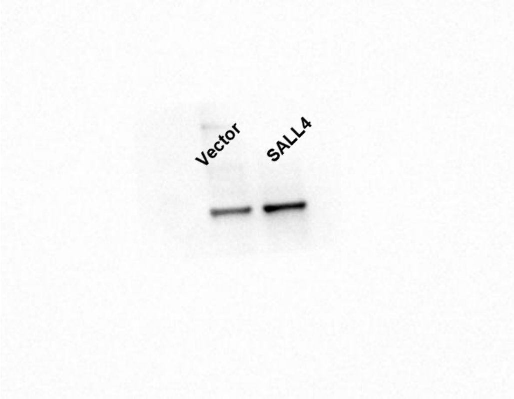

OE-SALL4-SOX2

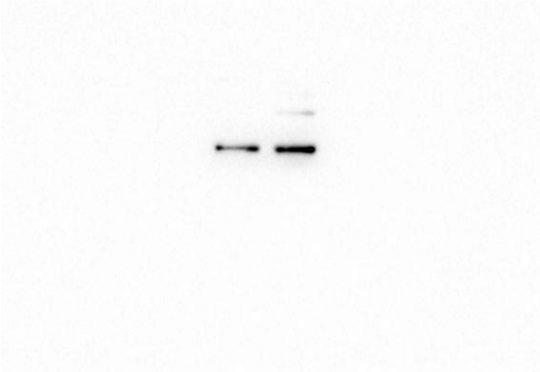

OE-SALL4-Nanog

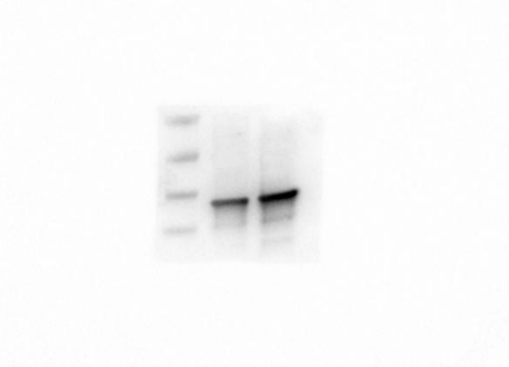

OE-SALL4-OCT4

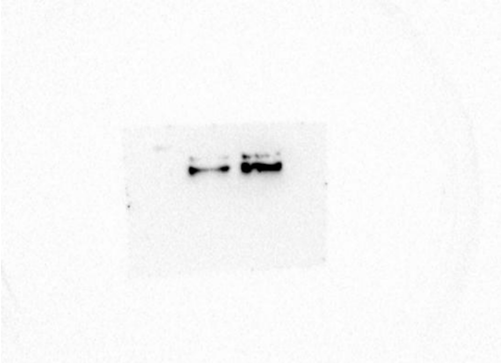

OE-SALL4-BMI1

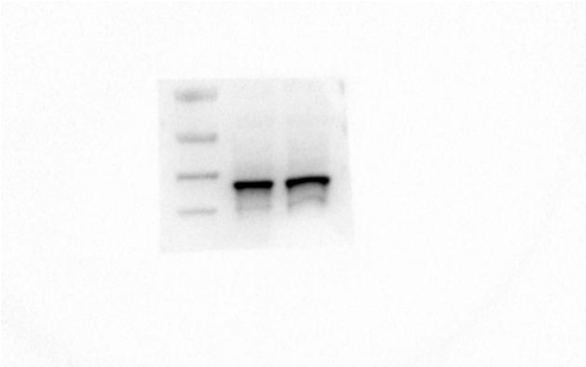

OE-SALL4-GAPDH

Figure4 F

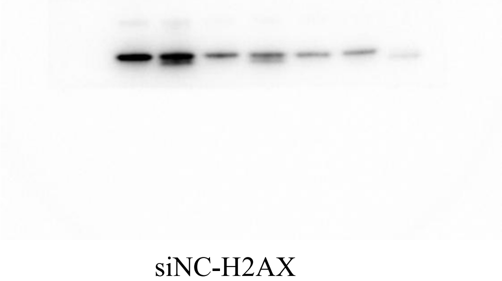

siNC-H2AX

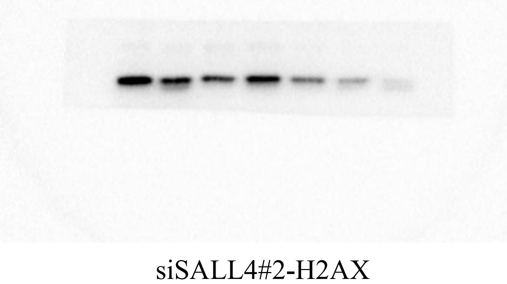

siSALL4#2-H2AX

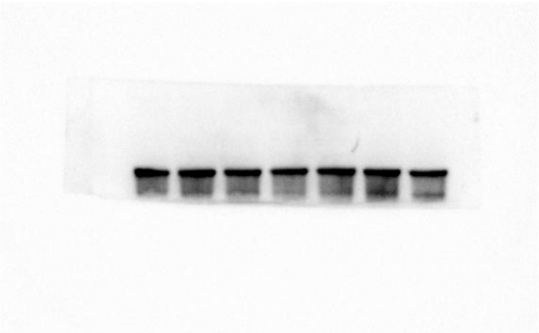

siNC-GAPDH

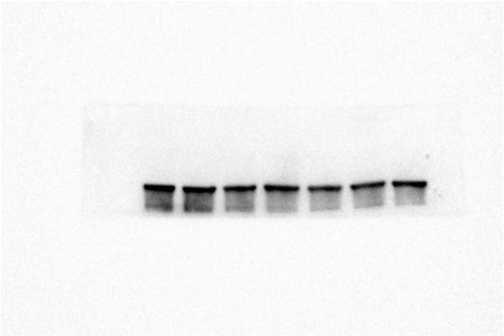

siSALL4#2-GAPDH

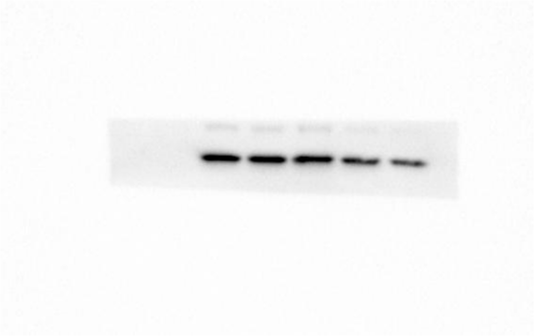

Vector-H2AX

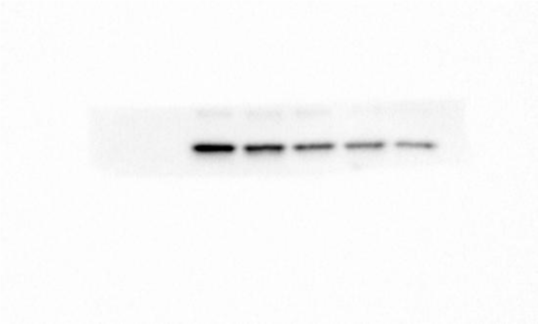

SALL4-H2AX

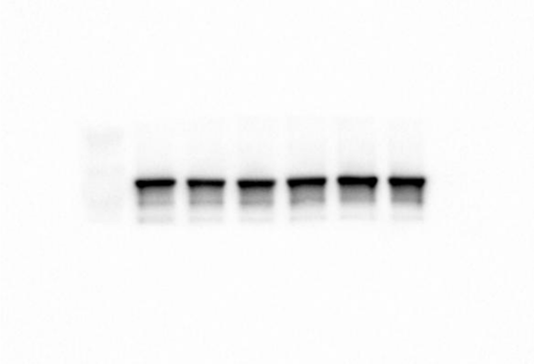

Vector-GAPDH

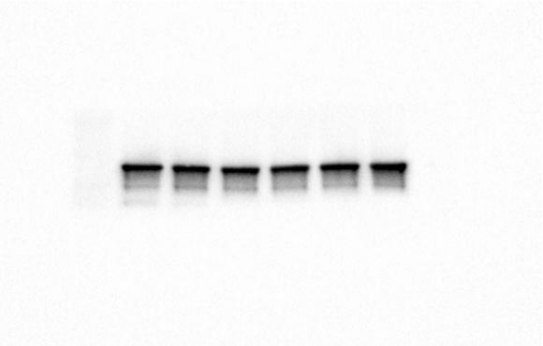

SALL4-GAPDH

Figure4 G

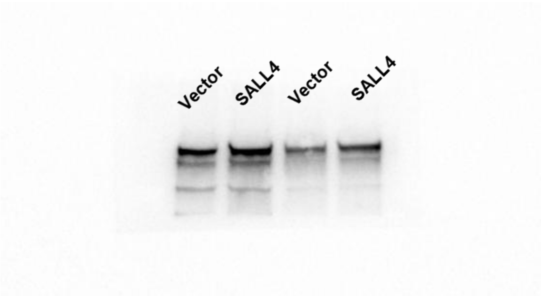

β-catenin

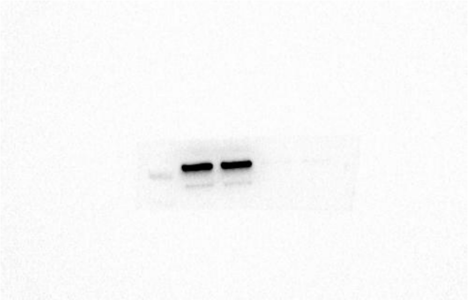

LaminB

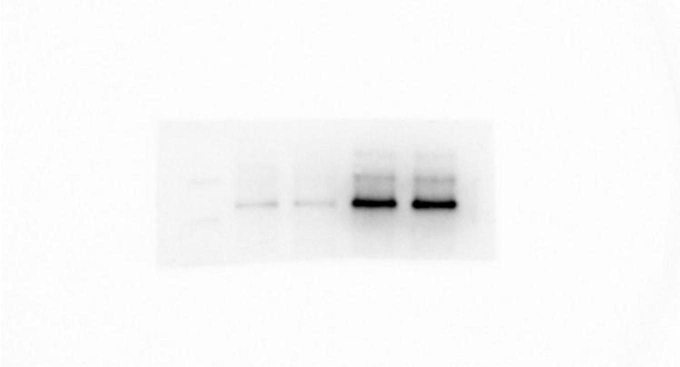

GAPDH

Figure4 I

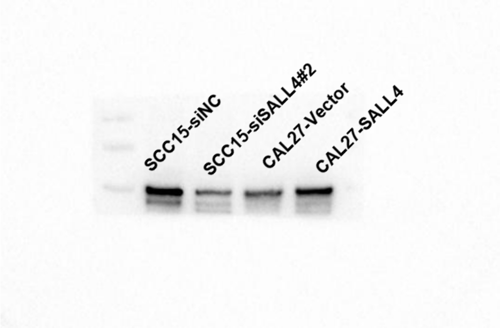

β-catenin

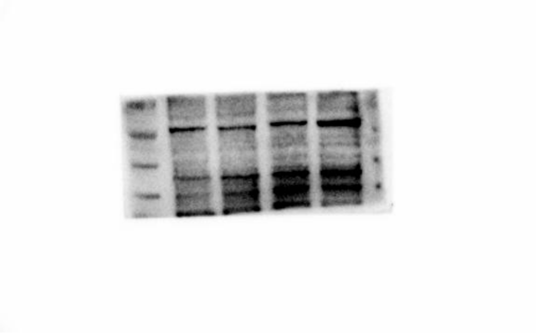

Wnt3a

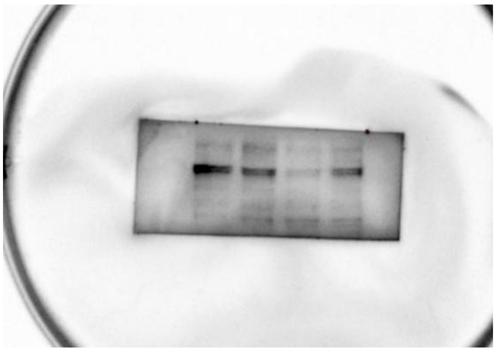

TCF1

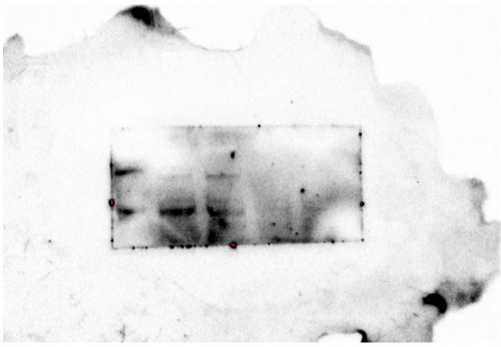

MMP9

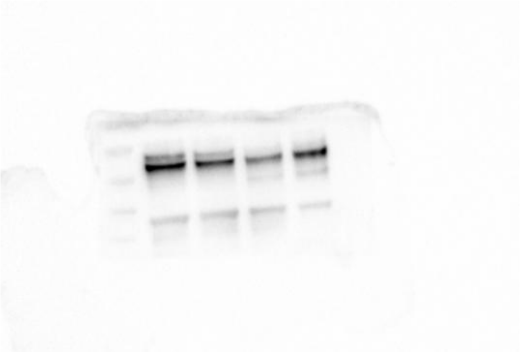

c-MYC

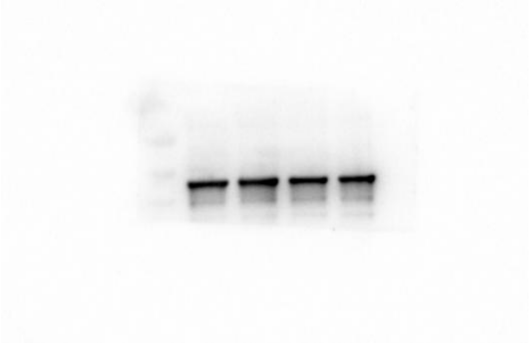

GAPDH

Figure4 J

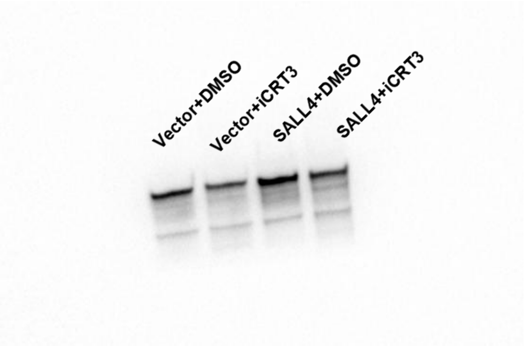

β-catenin

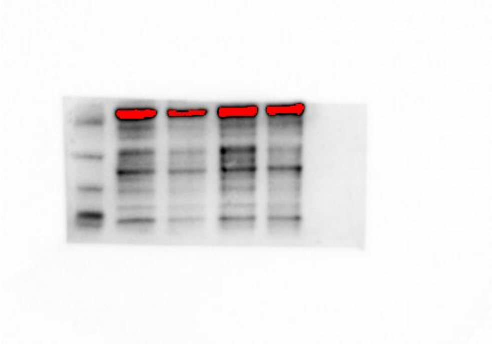

Wnt3a

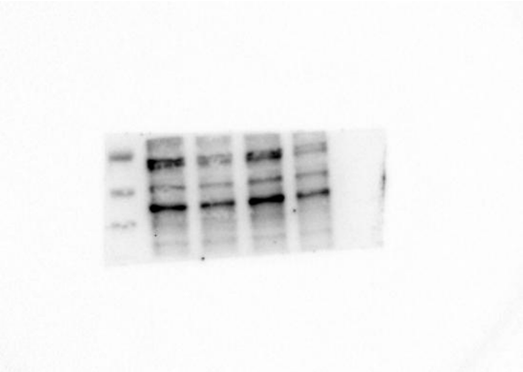

TCF1

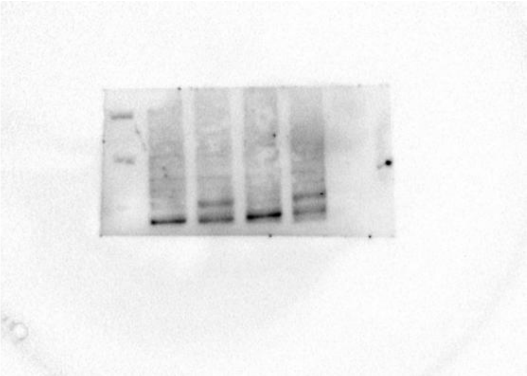

MMP9

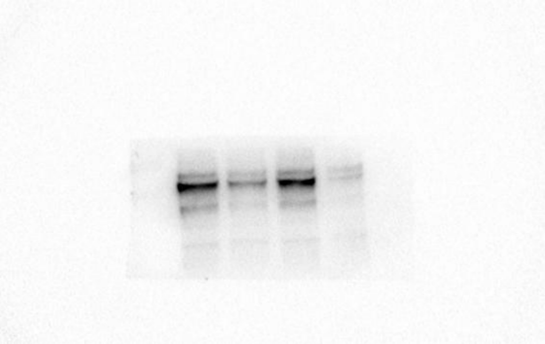

c-MYC

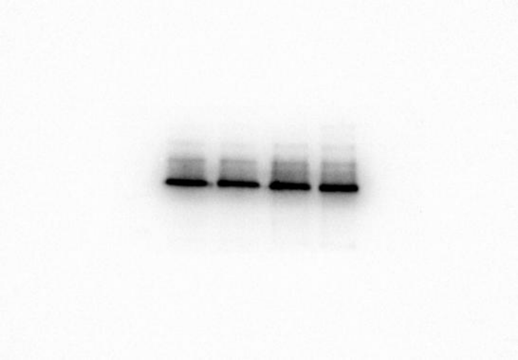

GAPDH

Figure5 E

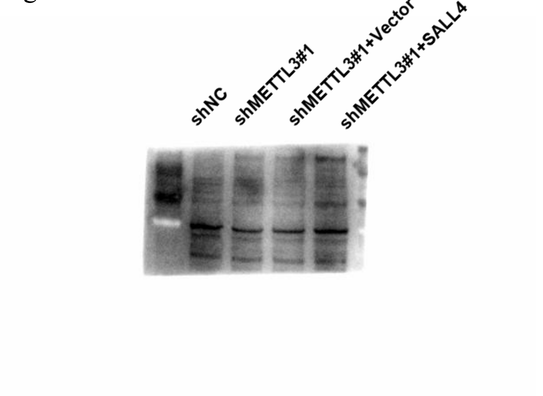

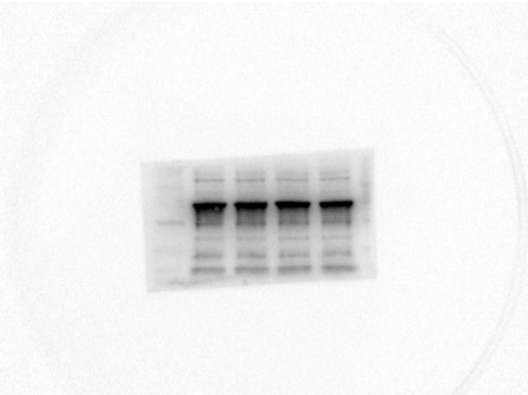

SALL4

GAPDH

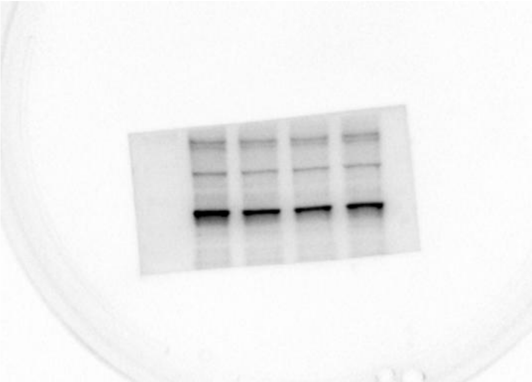

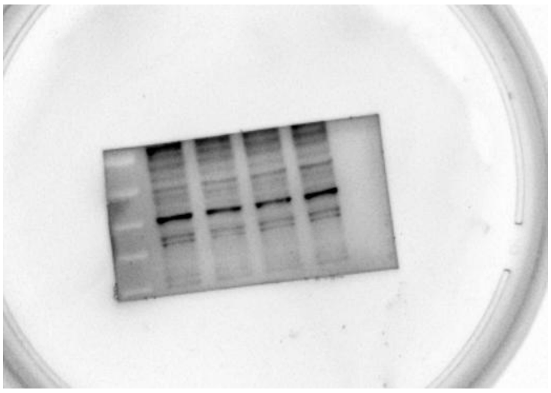

SOX2

Nanog

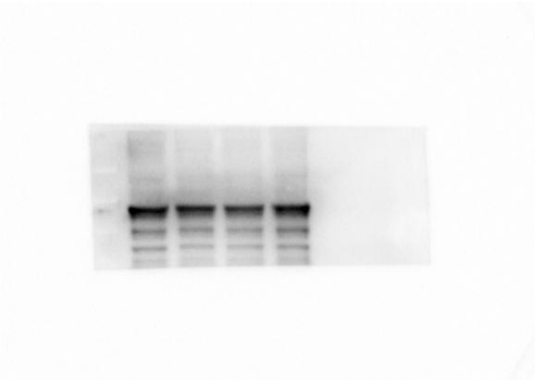

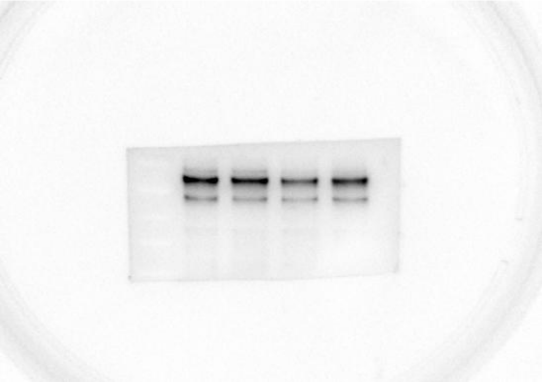

OCT4

c-MYC

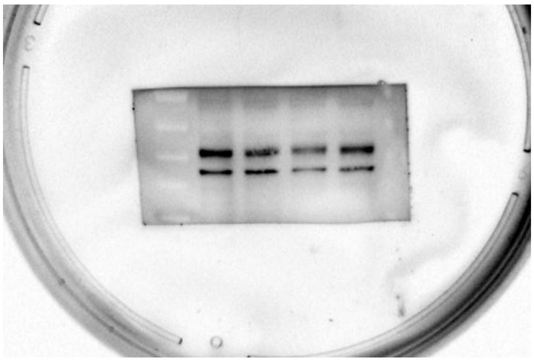

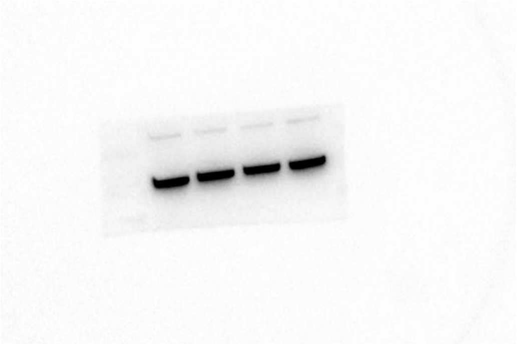

BMI1

GAPDH

Figure5 I

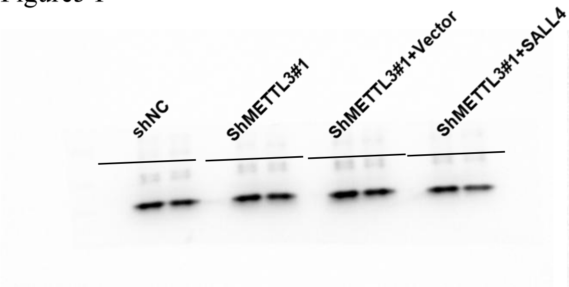

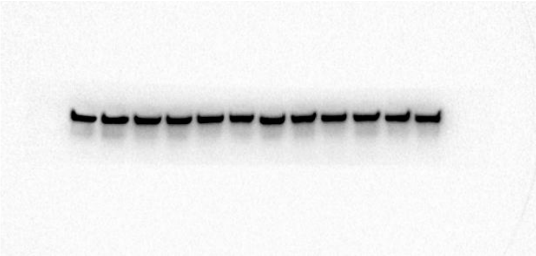

γ-H2AX

GAPDH

Supplementary Figure1 C

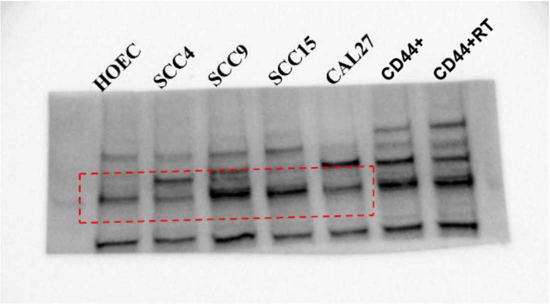

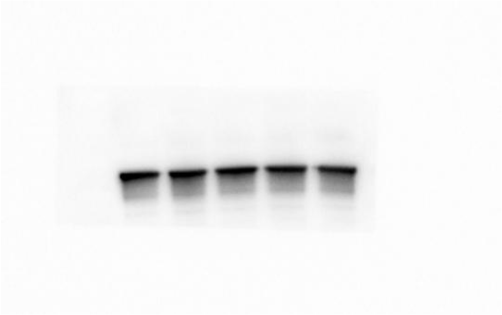

SALL4

GAPDH

Supplementary Figure2 D

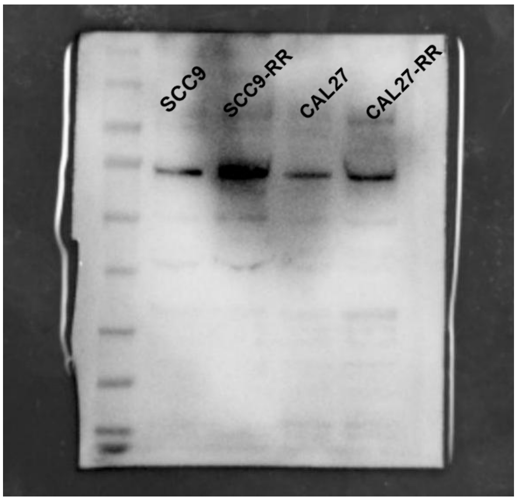

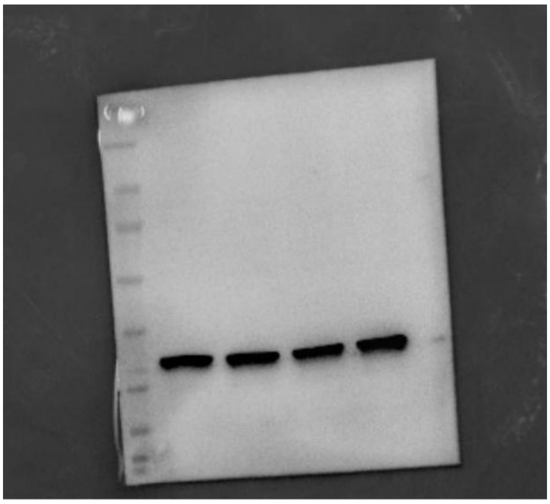

SALL4

GAPDH

Supplementary Figure2 E

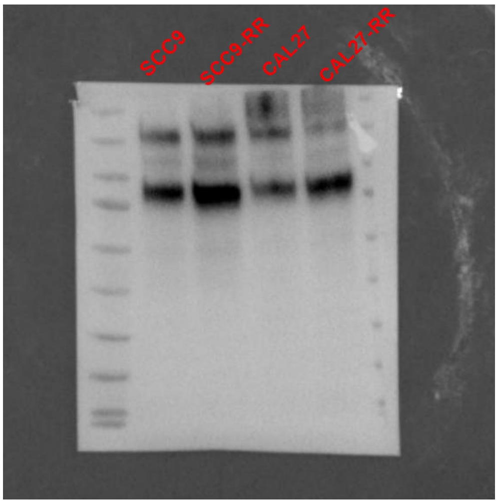

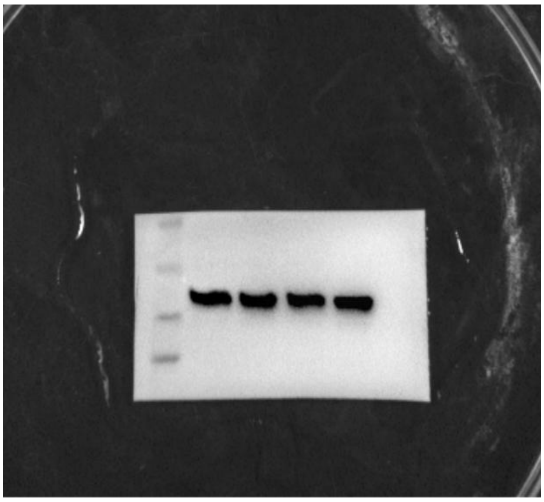

CD44

GAPDH

Supplementary Figure2 J

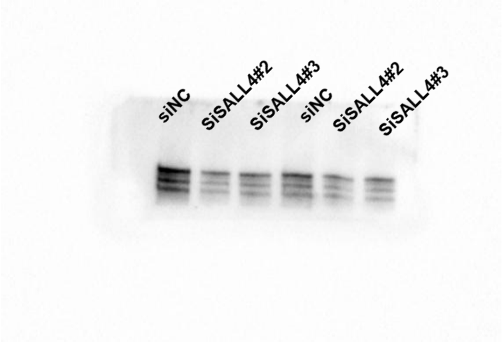

β-catenin

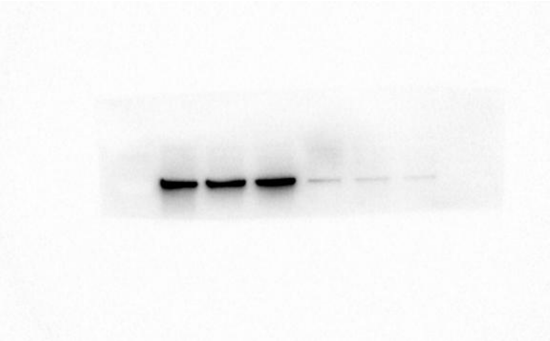

LaminB

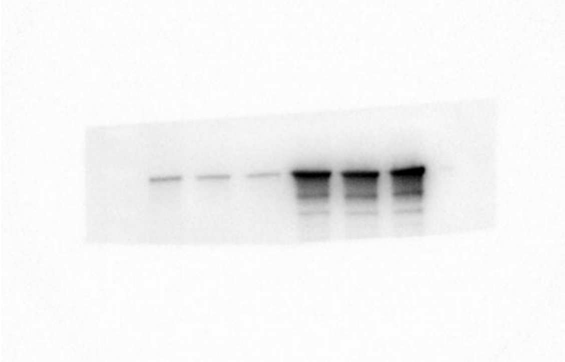

GAPDH
